# Supplementary material for: BrtB is an O-alkylating enzyme that generates fatty acid-bartoloside esters
Source: Nat Commun. 2020 Mar 19;11:1458. doi: 10.1038/s41467-020-15302-z (PMC7081238; doi:10.1038/s41467-020-15302-z)
Supplement: Supplementary file 1 — Supplementary Information [file 41467_2020_15302_MOESM1_ESM.pdf]

Supplementary Information

BrtB is an O-alkylating enzyme that generates fatty acid-bartoloside esters

Reis et al.

Index

	page
**Supplementary Figure 1** – Depletion of bartolosides in *Synechocystis salina* LEGE 06099 cells supplemented with fatty acids.	SI-4
**Supplementary Figure 2** – Formation of bartoloside esters upon supplementation of *S. salina* LEGE 06099 with (a) 5-hexynoic and (b) 6-heptynoic (**2**) acids.	SI-5
**Supplementary Note 1** – Structural elucidation of esterified bartolosides **3**, **4**, **7a** and **7b**.	SI-6
**Supplementary Figure 3** – Structural elucidation of **3** and **4**.	SI-10
**Supplementary Figure 4** – Bartoloside esters are formed in *S. salina* LEGE 06099 cells upon supplementation with fatty acids of different lengths and with the halogenated fatty acid 7-bromoheptanoate.	SI-11
**Supplementary Figure 5** – BrtB converts bartoloside A (**1**) and 6-heptynoic acid (**2**) into bartoloside esters *in vitro*.	SI-12
**Supplementary Figure 6** – BrtB converts bartoloside A (**1**) and palmitic acid into bartoloside esters *in vitro*.	SI-13
**Supplementary Figure 7 –** Substrate scope of BrtB.	SI-14
**Supplementary Figure 8 –** BrtB activity at different temperatures.	SI-15
**Supplementary Figure 9** – LC-HERSIMS/MS analysis of mono- and diesters of bartoloside A formed *in vitro* with either stable isotope labeled ([$^{18}O_2$]) or non-labeled acetate substrates.	SI-16
**Supplementary Figure 10** – LC-HRESIMS analysis of BrtB assays in D_2O-resuspended reaction buffer.	SI-17
**Supplementary Figure 11** – ^{1}H NMR- and HRESIMS/MS based assignment of the esterified alkyl chain in **7a** and **7b**.	SI-18
**Supplementary Figure 12** – Detection and HRESIMS/MS based structural assignment of natural fatty acid bartoloside monoesters produced by *S. salina* LEGE 06099.	SI-19
**Supplementary Figure 13** – Detection and HRESIMS/MS based structural assignment of natural fatty acid bartoloside esters produced by *S. salina* LEGE 06099.	SI-20
**Supplementary Figure 14** – Cytotoxicity assays against immortalized human cells.	SI-21
**Supplementary Figure 15** – Antimicrobial assays.	SI-22
**Supplementary Figure 16** – Selectivity of esterification reactions between **1** and palmitic acid.	SI-23
**Supplementary Figure 17** – Michaelis-Menten kinetic data for NStrep-BrtB.	SI-24
**Supplementary Figure 18** – Phylogeny of BrtB homologs and diversity of their associated biosynthetic gene clusters.	SI-25
**Supplementary Note 2 –** Protein and DNA sequences.	SI-26
**Supplementary Figure 19** – SDS-PAGE analysis of the Strep-NBrtB protein preparations, following purification and concentration.	SI-27

NMR spectroscopy data

**Supplementary Table 1 –** NMR Spectroscopic Data (^{1}H 400 MHz, ^{13}C 100 MHz, $CDCl_3$) for bartoloside A-17,29-diyl bis(hept-6-ynoate) (**3**).	SI-28
**Supplementary Figure 20** – ^{1}H NMR ($CDCl_3$, 400 MHz) spectrum of compound **3**.	SI-29
**Supplementary Figure 21** – ^{13}C NMR ($CDCl_3$, 100 MHz) spectrum of compound **3**.	SI-30
**Supplementary Figure 22** –HSQC ($CDCl_3$, 400 MHz) spectrum of compound **3**.	SI-31
**Supplementary Figure 23** –HMBC ($CDCl_3$, 400 MHz) spectrum of compound **3**.	SI-32

Supplementary Figure 24 –COSY ($CDCl_3$, 400 MHz) spectrum of compound **3**. SI-33

Supplementary Figure 25 – HRESIMS spectrum of compound **3**. SI-34

Supplementary Table 2 – NMR Spectroscopic Data (^{1}H 400 MHz, ^{13}C 100 MHz, $CDCl_3$) for bartoloside G-17-yl hept-6-ynoate (**4**). SI-35

Supplementary Figure 26 – ^{1}H NMR ($CDCl_3$, 400 MHz) spectrum of compound **4**. SI-36

Supplementary Figure 27 – ^{13}C NMR (APT, $CDCl_3$, 100 MHz) spectrum of compound **4**. SI-37

Supplementary Figure 28 – HSQC ($CDCl_3$, 400 MHz) spectrum of compound **4**. SI-38

Supplementary Figure 29 – HMBC ($CDCl_3$, 400 MHz) spectrum of compound **4**. SI-39

Supplementary Figure 30 – COSY ($CDCl_3$, 400 MHz) spectrum of compound **4**. SI-40

Supplementary Figure 31 – HRESIMS spectrum of compound **4**. SI-41

Supplementary Table 3 – NMR Spectroscopic Data (^{1}H 400 MHz, ^{13}C 100 MHz, $CDCl_3$) for bartoloside A-17-yl palmitate (**7a**). SI-42

Supplementary Figure 32 – ^{1}H NMR ($CDCl_3$, 400 MHz) spectrum of compound **7a**. SI-43

Supplementary Figure 33 – ^{13}C NMR (APT, $CDCl_3$, 100 MHz) spectrum of compound **7a**. SI-44

Supplementary Figure 34 – HSQC ($CDCl_3$, 400 MHz) spectrum of compound **7a**. SI-45

Supplementary Figure 35 – HMBC ($CDCl_3$, 400 MHz) spectrum of compound **7a**. SI-46

Supplementary Figure 36 – COSY ($CDCl_3$, 400 MHz) spectrum of compound **7a**. SI-47

Supplementary Figure 37 – HRESIMS spectrum of compound **7a**. SI-48

Supplementary Table 4 – NMR Spectroscopic Data (^{1}H 600 MHz, ^{13}C 100 MHz, $CDCl_3$) for partially purified bartoloside A-29-yl palmitate (**7b**). SI-49

Supplementary Figure 38 – ^{1}H NMR ($CDCl_3$, 600 MHz) spectrum of partially purified compound **7b**. SI-50

Supplementary Figure 39 – ^{13}C NMR (APT, $CDCl_3$, 150 MHz) spectrum of partially purified compound **7b**. SI-51

Supplementary Figure 40 – HRESIMS spectrum of compound **7b**. SI-52

Supplementary references SI-53

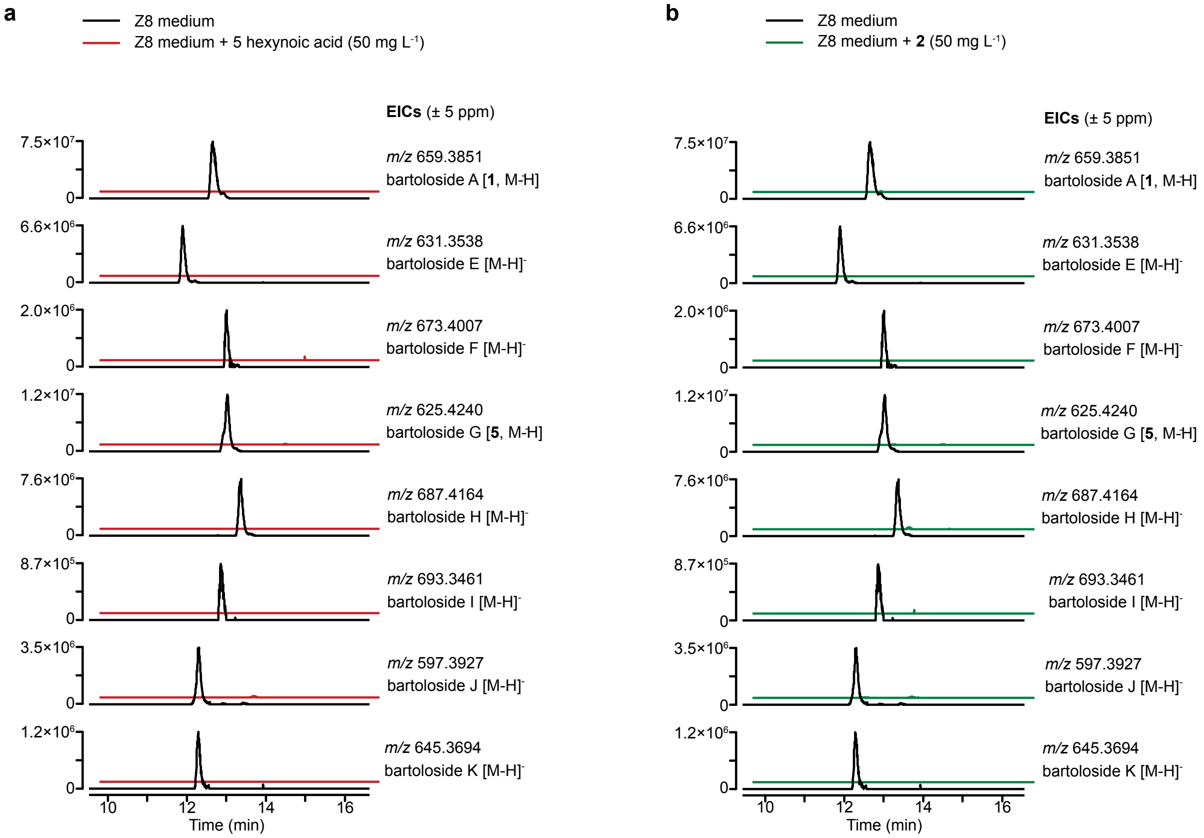

Supplementary Figure 1 – Depletion of bartolosides in *Synechocystis salina* LEGE 06099 cells supplemented with fatty acids. LC-HRESIMS analysis of crude extracts from *S. salina* LEGE 06099 cells shows that supplementation of cultures of this cyanobacterium with 5-hexynoic (a) or 6-heptynoic (**2**) (b) acids, at 50 mg L^{-1}, leads to cellular depletion of bartolosides.

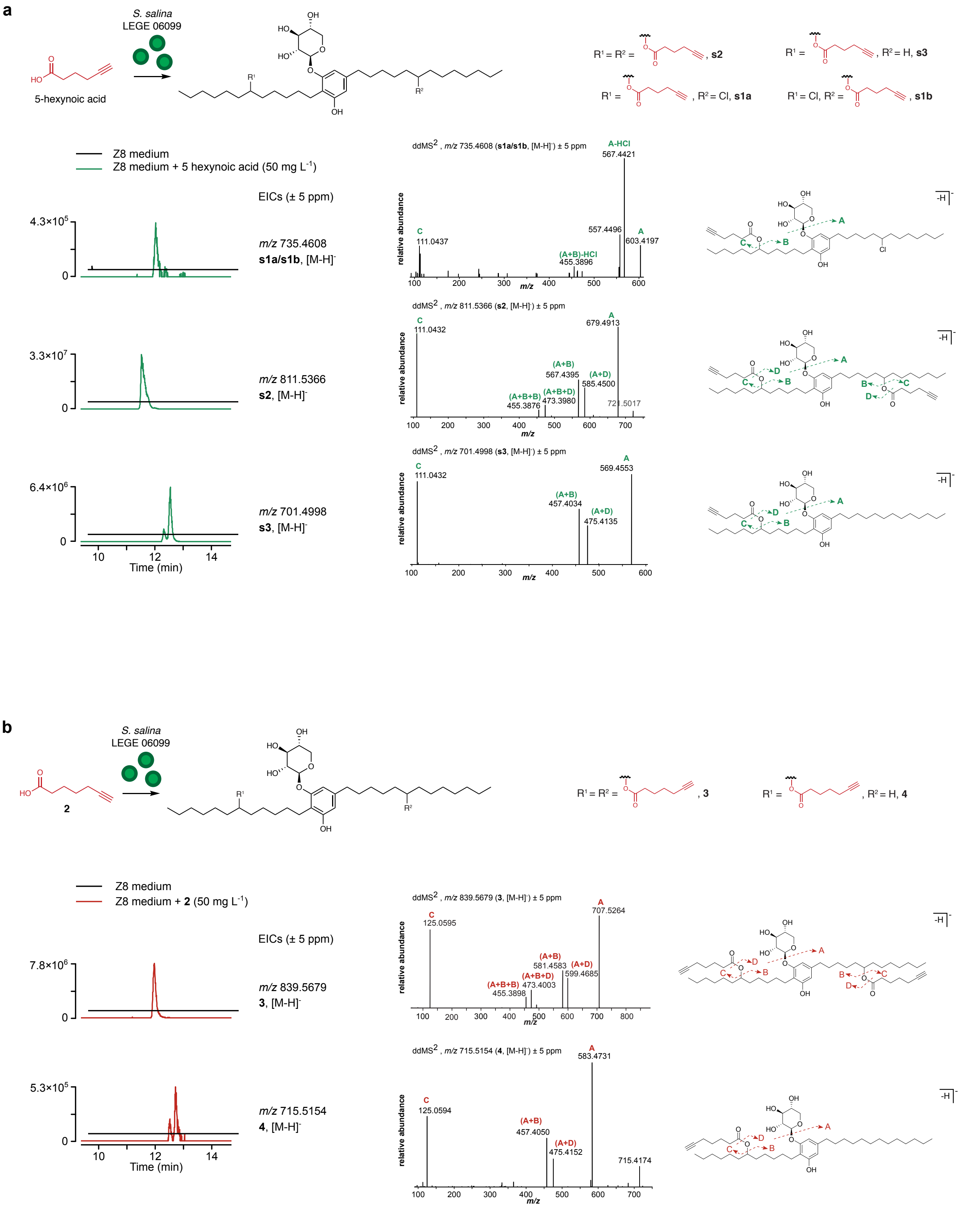

Supplementary Figure 2 – Formation of bartoloside esters upon supplementation of *S. salina* LEGE 06099 with (a) 5-hexynoic and (b) 6-heptynoic (**2**) acids. Shown are proposed structures and respective extracted ion chromatograms (EICs) for the esters produced after supplementation with each of the fatty acids, as well as the annotated LC-HRESIMS/MS data for compounds **s1a**/**s1b**, **s2**, **s3**, **3** and **4**.

Supplementary Note 1 – Structural elucidation of esterified bartolosides **3**, **4**, **7a** and **7b**

*bartoloside A-17,29-diyl bis(hept-6-ynoate) (**3**)*

HRESIMS analysis of compound **3** showed a $[M-H]^-$ peak at *m/z* 839.5685, compatible with a molecular formula of $C_{50}H_{80}O_{10}$ (calcd for $C_{50}H_{79}O_{10}^-$, *m/z* 839.56867) and 11 degrees of unsaturation. The ^1H and ^{13}C NMR spectra showed typical bartoloside resonances, namely for the aromatic ring (δ_C 155.8, 154.6, 141.9, 116.3, 110.4 and 107.8) and xylosyl (δ_C 101.4, 72.6, 74.9, 69.7 and 64.5) (Supplementary Table 1). Characteristic resonances for two ester/acid carbonyls (δ_C 174.1 and 173.9) and two alkyne moities (δ_C 68.8, δ_H 1.95) were also observed. The HRESIMS-derived molecular formula and isotope pattern of **3** dictated the absence of halogens in the molecule. The molecular formula had an additional $C_{14}H_{18}O_4$ when compared to the major bartoloside **1** produced by *S. salina* LEGE 06099 (bartoloside A) consistent with the incorporation of two 6-heptynoic acid moieties. This initial analysis strongly suggested that compound **3** could correspond to a bartoloside derivative where the chlorinated positions in **1** were substituted by the supplemented 6-heptynoic acid (**2**). To obtain further insights regarding the structure of **3**, we performed 2D NMR experiments (HSQC, HMBC, COSY). Correlational data for the glycosylated dialkylresorcinol moiety were highly similar to those of the bartolosides and it was also possible to deduce the structures of two intact 6-heptynoate spin systems from COSY and HMBC (Supplementary Fig. 3). Notably, two conspicuous protons resonating at δ_H 4.87 (H-17 and H-29) in **3** are not found in any of the previously reported bartolosides. Each of these protons was positioned in the middle of an alkyl chain (Supplementary Fig. 3) and showed HMBC correlations to the carbonyls C-1' (δ_C 174.1) and C-1'' (δ_C 173.9). Strong deshielding indicated that these were oxymethine protons and established the directionality of the ester linkages. HRESIMS/MS data, which show fragments corresponding to 6-heptynoate (*m/z* 125.05 $[M-H]^-$, Supplementary Fig. 2) are in accordance with this observation. Additionally, the resonances for the chlorinated carbons in bartoloside A (δ_C 64.9, δ_H 4.01)[1,2] are not present in **3**, further implying ester linkages in positions 17 and 29. While it was not possible to fully connect the oxymethines to the dialkylresorcinol rings, the HRESIMS/MS data of in-source fragments of **3** (Supplementary Fig. 3) matches the data for bartoloside A (**1**) and other bartolosides in our previous structural elucidation efforts.[1,2] As such, we propose that the esterified positions in **3** correspond to the chlorinated positions in **1**.

bartoloside G-17-yl hept-6-ynoate (4)

HRESIMS data of compound **4** showed a prominent peak at *m/z* 715.5161 $[M-H]^-$ which was compatible with a molecular formula of $C_{43}H_{72}O_8$ (calcd for $C_{43}H_{71}O_8^-$, *m/z* 715.5162) and 8 degrees of unsaturation. The ^1H and ^{13}C NMR data (Supplementary Table 2) were highly similar to those from compound **3**, notably featuring a carbonyl resonance (δ_C 174.1) and an alkyne moiety signal (δ_C 68.8, δ_H 1.95). Additionally, the typical bartoloside resonances for the aromatic ring (δ_C 155.7, 154.3, 142.5, 116.1, 110.4, 108.1) and xylosyl group (δ_C 101.4, 72.6, 74.9, 69.7 and 64.5) were present. The HRESIMS-derived molecular formula and isotope pattern of **4** indicated the absence of halogens in the molecule and an additional $C_7H_9O_2$ when compared to another bartoloside produced by *S. salina* LEGE 06099 (bartoloside G, **5**). This would be consistent with **4** resulting from the incorporation of one 6-heptynoic acid moiety into **5**. Two-dimensional NMR experiments (HSQC, HMBC and COSY) showed the presence of an intact 6-heptynoate-derived spin system from COSY and HMBC correlations (Supplementary Fig. 3). The HMBC correlations for H-17 (δ_H 4.88) with C-1' (δ_C 174.1) indicated an identical ester linkage to that of **3**, at the C-17 (δ_C 74.7) position (Supplementary Fig. 3). In line with these observations, the HRESIMS/MS data for **4** showed a 6-heptynoate-derived fragment (*m/z* 125.05 $[M-H]^-$, Supplementary Fig. 2). By isolating and carrying out HRESIMS/MS analysis on an in-source fragment of **4**, we obtained diagnostic fragments indicating a fully aliphatic alkyl chain with 13 carbons (*m/z* 249.22 and *m/z* 291.23, Supplementary Fig. 3), as well as fragments consistent with an alcohol group in the C12 alkyl chain (e.g. *m/z* 225.18 and *m/z* 319.23, Supplementary Fig. 3). We thus propose that metabolite **4** is a derivative of the naturally occurring **5**, in which the chlorinated position is substituted by a 6-heptynoate moiety.

*bartoloside A-17-yl palmitate (7**a**)*

HRESIMS performed for compound **7a** presented a peak with *m/z* 879.6504 $[M-H]^-$ (calcd for $C_{52}H_{92}O_8Cl^-$ 879.6499), compatible with a molecular formula of $C_{52}H_{93}O_8Cl$ and 6 degrees of unsaturation. The ^1H and ^{13}C NMR data indicated the presence of the characteristic aromatic resorcinol ring and xylosyl group observed in bartolosides from *S. salina* LEGE 06099[2] (Supplementary Table 3). A carbonyl resonance was also present (δ_C 174.7) as observed for bartoloside esters **3** and **4**. The additional $C_{16}H_{31}O_2$ (compared to **1**) was consistent with the esterification of one palmitic acid moiety with bartoloside A (**1**) and the concomitant loss of one chlorine atom (as seen for **3** and **4**). Support to this hypothesis was gained from HRESIMS/MS analysis of the in-source-formed species with *m/z* 473.4003[2], which showed diagnostic fragments that are consistent with a backbone similar to that of **1** (Fig. 3), as well as from 2D NMR experiments: an HSQC correlation between δ_H 3.88 and δ_C 64.5, likely corresponding to a chlorinated methine, was part of a spin system that expanded, on both directions into degenerate CH_2 resonances, indicated that this corresponded to a mid-chain chlorination, characteristic of the bartolosides. HRESIMS/MS data was consistent with the presence of a palmitate-derived fragment (*m/z* 255.23 $[M-H]^-$, Fig. 3). The 1D and 2D NMR data showed an additional methyl group (compared to **1**) at the terminal position of an alkyl chain, as well as a bigger CH_2 envelope, both in line with the presence of a palmitate moiety in compound **7a**. The oxymethine proton resonating at δ_H 4.88 presented HMBC correlations to C-1' (δ_C 174.7) in the palmitate moiety and was itself part of another spin system that degenerated into CH_2 envelopes; hence it was likely positioned in the middle of the non-chlorinated alkyl chain. All these observations supported our proposal of **7a** being a palmitate ester of **1**. Because **1** has two halogenated methine moieties, to clarify which chain contained the esterification in **7a**, we compared the ^1H NMR spectra for **7a** with the previously reported[2] data for **1**. Interestingly, we found that the multiplicity of H_2-12 (δ_H 2.58) changed from a complex multiplet in bartoloside A to a triplet in **7a**, while the multiplicity of H_2-24 (δ_H 2.50) remained unaffected (Supplementary Fig. 11). As such, we propose that position 17, in the same alkyl chain as the H_2-12 benzylic protons, is esterified in **7a**. HRESIMS/MS analysis is consistent with this assignment (Supplementary Fig. 11).

*bartoloside A-29-yl palmitate (7**b**)*

LC-HRESIMS showed a major peak with *m/z* 879.6497 $[M-H]^-$ (calcd for $C_{52}H_{92}O_8Cl$), consistent with a bartoloside A monopalmitate. HRESIMS/MS data confirmed the presence of a palmitate-derived fragment (*m/z* 255.23 $[M-H]^-$) as observed for the in vitro formed **7a/7b** co-eluting mixture (Supplementary Fig. 6). The ^1H and ^{13}C NMR data (Supplementary Table 4) indicated the presence of the characteristic resonances for the aromatic resorcinol ring and xylosyl group observed in bartolosides from *S. salina* LEGE06099.[2] A carbonyl resonance at δ_C 174.5 was present as previously verified for bartoloside esters **3**, **4** and **7a**. Both the ^1H and ^{13}C NMR data were highly similar to those of **7a**. To clarify which chain contained the esterification in **7b**, we compared the ^1H NMR spectra for this subfraction with the one from **7a** and the previously reported[2] data for **1**. We found that the H_2-12 (δ_H 2.59) protons resonated as a multiplet, as observed also for bartoloside A, whereas in **7a** these protons show up as a triplet (Supplementary Fig. 11). HRESIMS/MS data of the in-source formed fragment with *m/z* 473.4002 $[M-H]^-$ (dialkylresorcinol core and one secondary alcohol derived from the ester) revealed a fragment with *m/z* 265.2185 $[M-H]^-$, confirming the presence of the ester in the C_{13} alkyl chain, i.e. the C-5 substituent (Supplementary Fig. 11). Therefore, we propose that **7b** corresponds to a bartoloside A monopalmitate with the ester positioned at C-29.

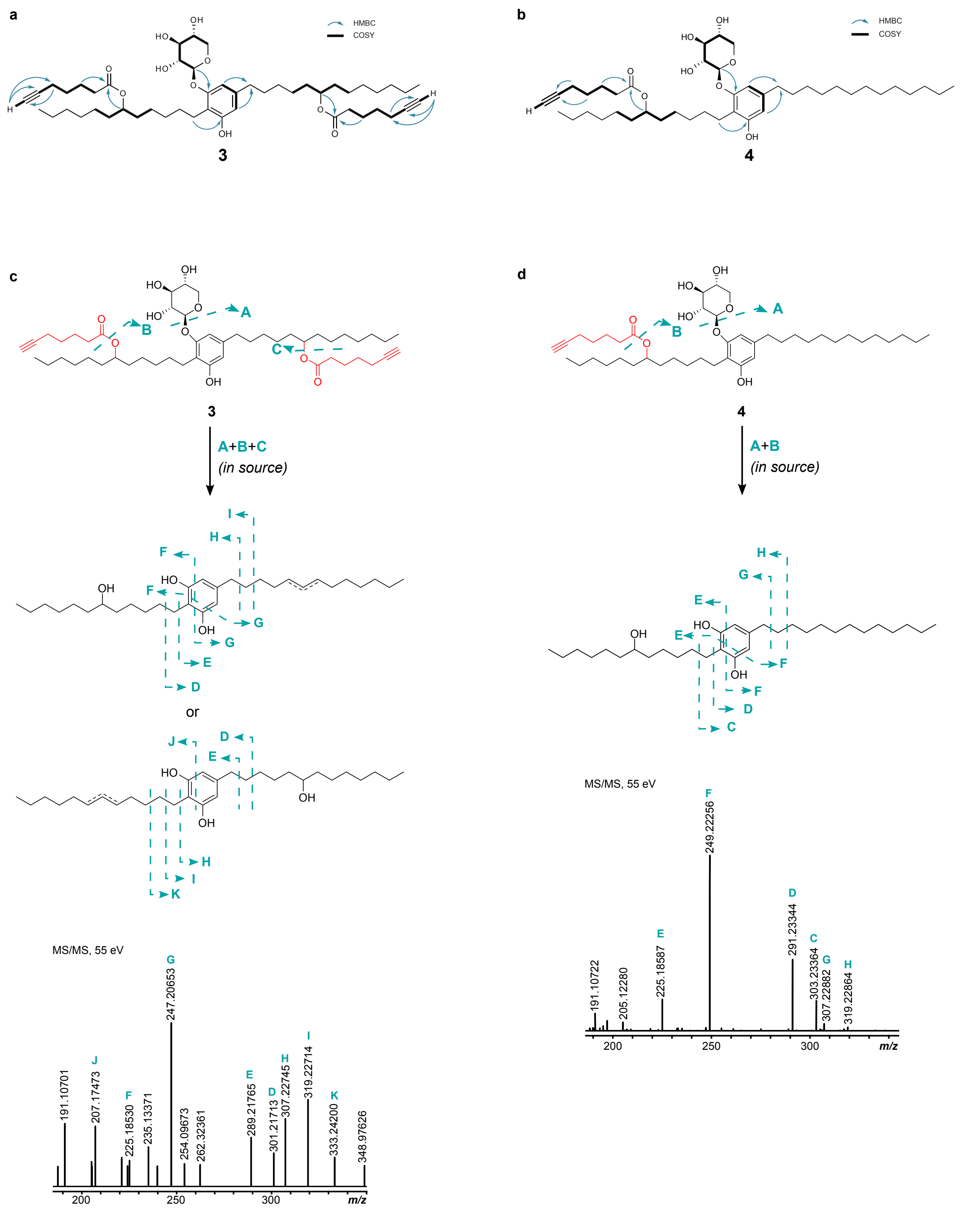

Supplementary Figure 3 – Structural elucidation of **3** and **4**. Key HMBC and COSY correlations for compounds **3** (a) and **4** (b) and annotated HRESIMS/MS spectra for an in-source generated fragment of **3** (c) and **4** (d), providing structural insight into the alkyl substitution pattern of the resorcinol ring.

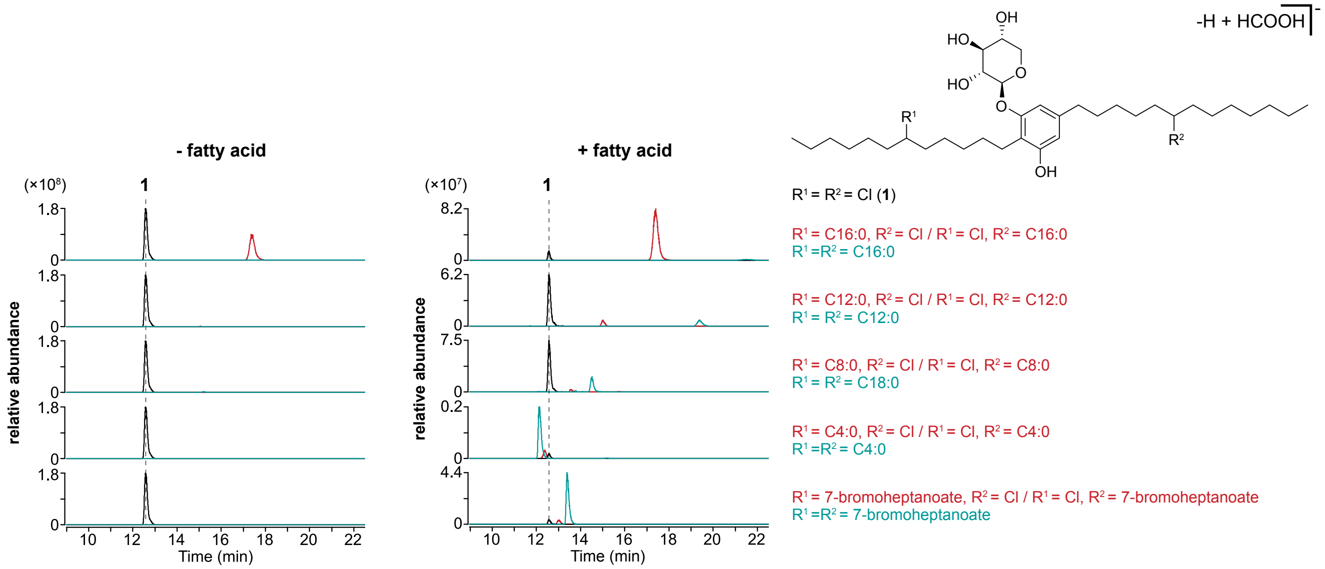

Supplementary Figure 4 – Bartoloside esters are formed in *S. salina* LEGE 06099 cells upon supplementation with fatty acids of different lengths and with the halogenated fatty acid 7-bromoheptanoate. Depticted are LC-HRESIMS extracted ion chromatograms (EICs), in non-supplemented (left panel) and supplemented (right panel) cultures of the cyanobacterium, for the [M-H+HCOOH]$^-$ adduct of the proposed bartoloside A esters.

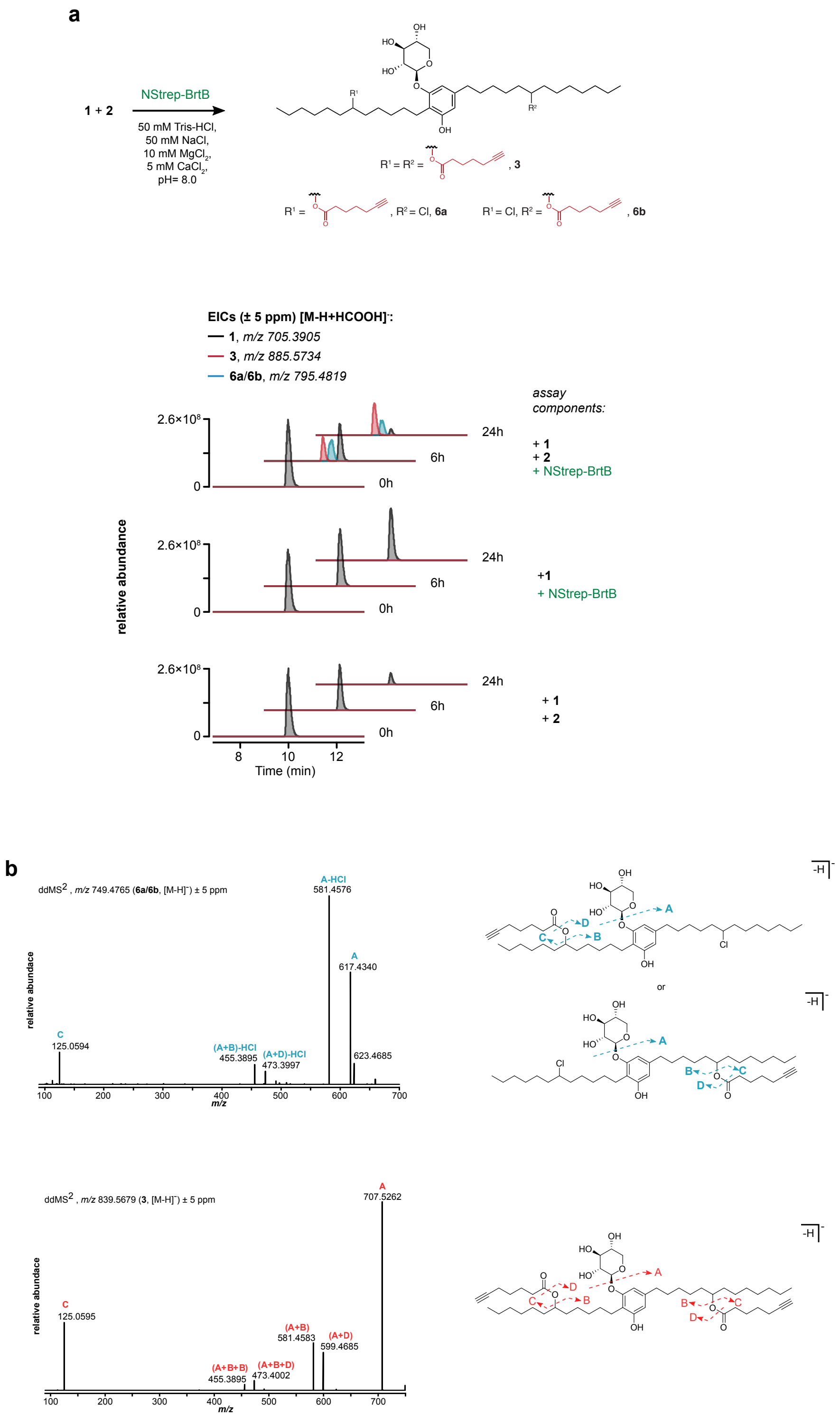

Supplementary Figure 5 – BrtB converts bartoloside A (**1**) and 6-heptynoic acid (**2**) into bartoloside esters *in vitro*. **a**) Extracted Ion Chromatograms (EICs) obtained from LC-HRESIMS analysis of a full assay with NStrep-BrtB, and control assays without either NStrep-BrtB or fatty acid **2**. **b**) LC-HRESIMS/MS analysis of the monoester(s) and diester formed in the full assay.

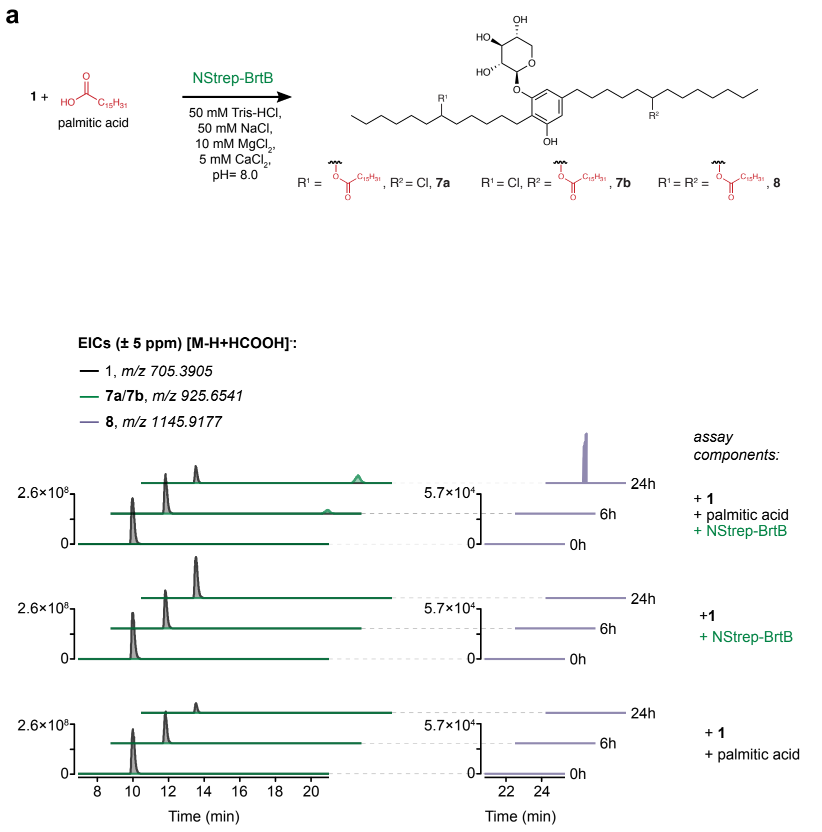

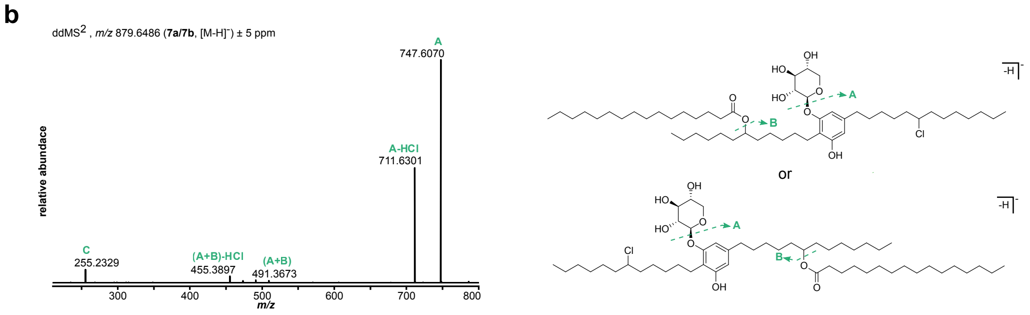

Supplementary Figure 6 – BrtB converts bartoloside A (**1**) and palmitic acid into bartoloside esters *in vitro*. **a**) Extracted Ion Chromatograms (EICs) obtained from LC-HRESIMS analysis of a full assay with NStrep-BrtB, and control assays without either NStrep-BrtB or palmitic acid. **b**) LC-HRESIMS/MS analysis of the bartoloside ester **7a**/**7b** products formed in the full assay (it was not possible to fragment the diester **8** due to low abundance of the precursor ion).

a

electrophile + nucleophile —NStrep-BrtB (20 mM HEPES, 50 mM NaCl, 10 mM $MgCl_2$, 5 mM $CaCl_2$, pH= 8.0)→ monoesters + diesters

nucleophile	electrophile	% conversion (monoesters+diester) assay #1	assay #2
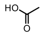	bartoloside A (**1**)	1.5	1.7
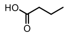	bartoloside A (**1**)	9.0	8.2
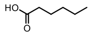	bartoloside A (**1**)	79.4	66.4
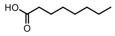	bartoloside A (**1**)	65.7	76.9
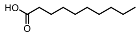	bartoloside A (**1**)	72.6	78.0
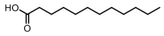	bartoloside A (**1**)	52.6	62.1
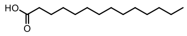	bartoloside A (**1**)	51.4	52.1
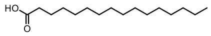	bartoloside A (**1**)	33.9	26.5
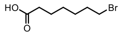	bartoloside A (**1**)	25.6	26.9
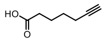	bartoloside A (**1**)	71.4	45.7
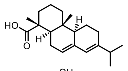	bartoloside A (**1**)	n.d.	n.d.
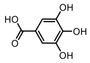	bartoloside A (**1**)	n.d.	n.d.
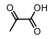	bartoloside A (**1**)	n.d.	n.d.
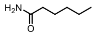	bartoloside A (**1**)	n.d.	n.d.
	bartoloside A (**1**)	n.d.	n.d.
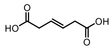	bartoloside A (**1**)	n.d.	n.d.
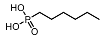	bartoloside A (**1**)	n.d.	n.d.
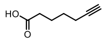	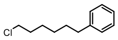	n.d.	n.d.
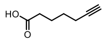	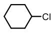	n.d.	n.d.
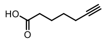		n.d.	n.d.
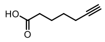	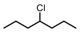	n.d.	n.d.

n.d. - not detected

b

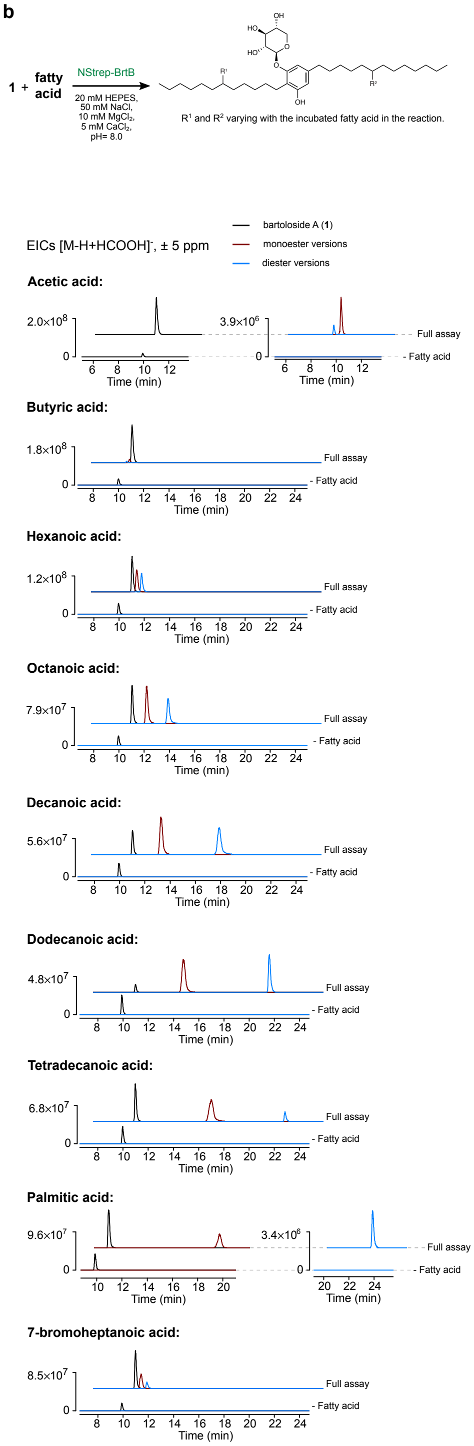

Supplementary Figure 7 – Substrate scope of BrtB. (a) Estimated conversions (EIC peak areas in LC-HRESIMS analysis, assuming similar ionization behavior) in 10 h assays using different substrates. (b) Illustrative LC-HRESIMS analysis of substrate scope assays (one out of two performed) in which conversion to products was observed. Assays contained 1 µM recombinant NStrep-BrtB, 100 µM electrophile and 200 µM nucleophile (except for reactions with monochlorinated electrophiles, in which a 100 µM nucleophile concentration was used).

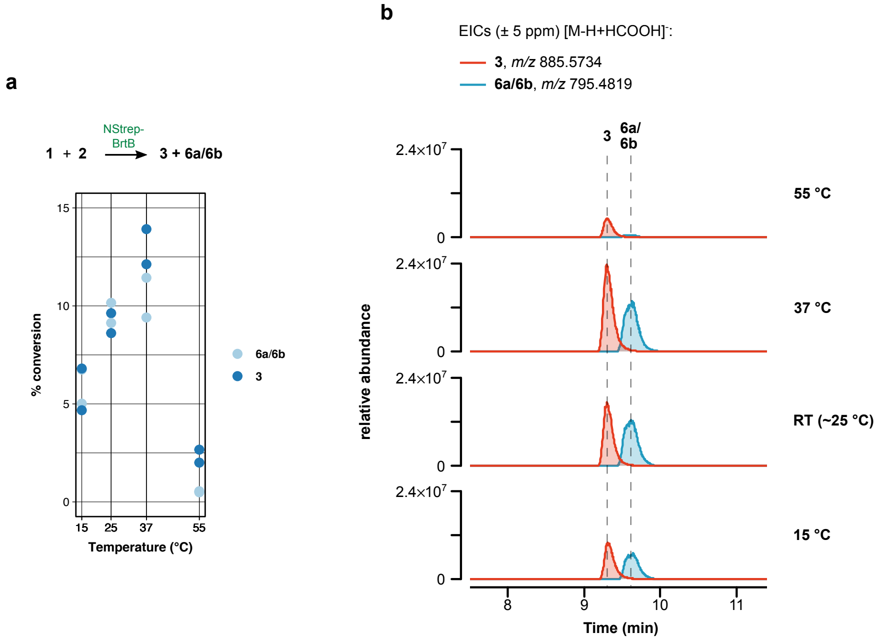

Supplementary Figure 8 – BrtB activity at different temperatures. (a) Estimated conversions (EIC peak areas in LC-HRESIMS analysis, assuming similar ionization behavior) in 24 h assays at different temperatures. (b) Representative LC-HRESIMS data obtained in the assays.

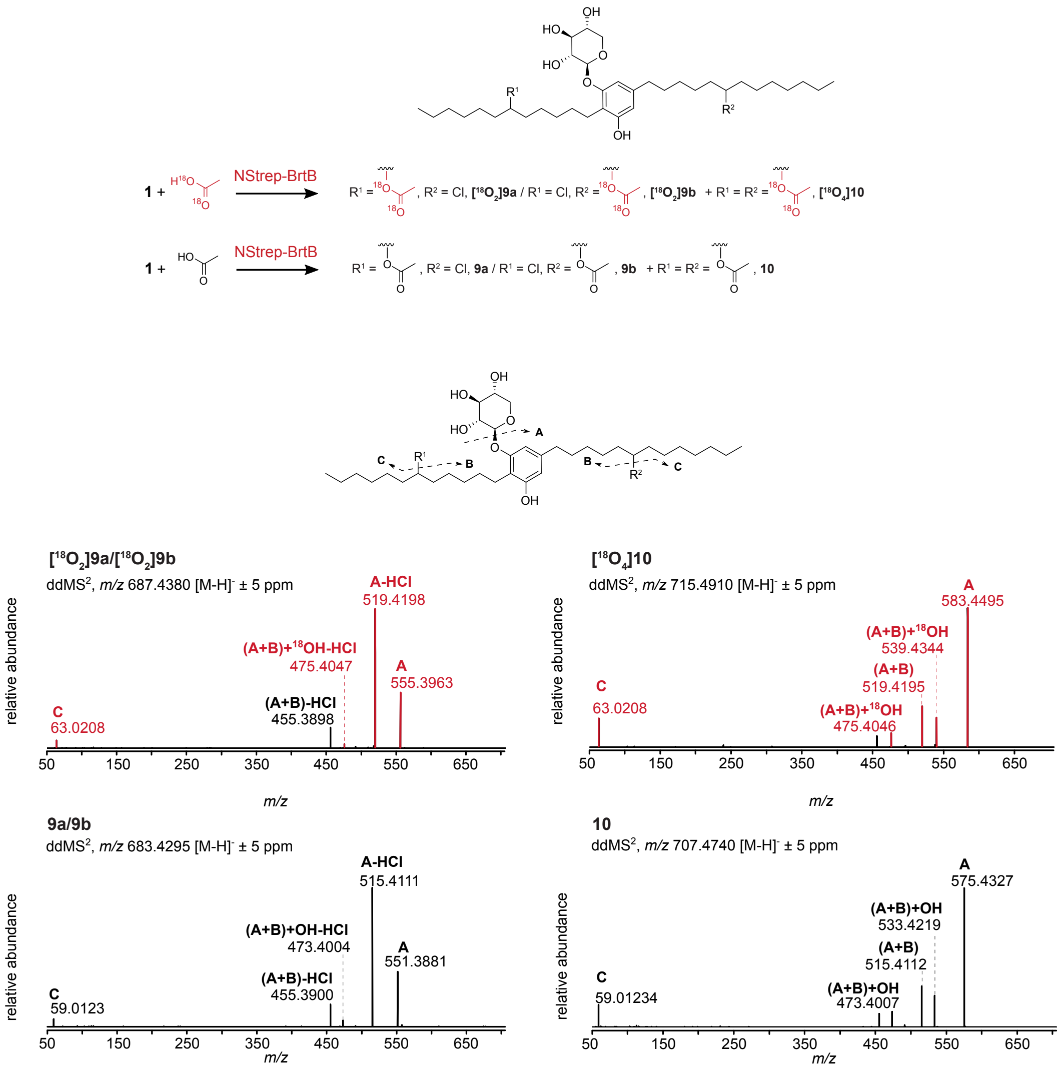

Supplementary Figure 9 – LC-HERSIMS/MS analysis of mono- and diesters of bartoloside A formed *in vitro*, following 24 h incubations with either stable isotope labeled ([$^{18}O_2$]) or non-labeled acetate substrates.

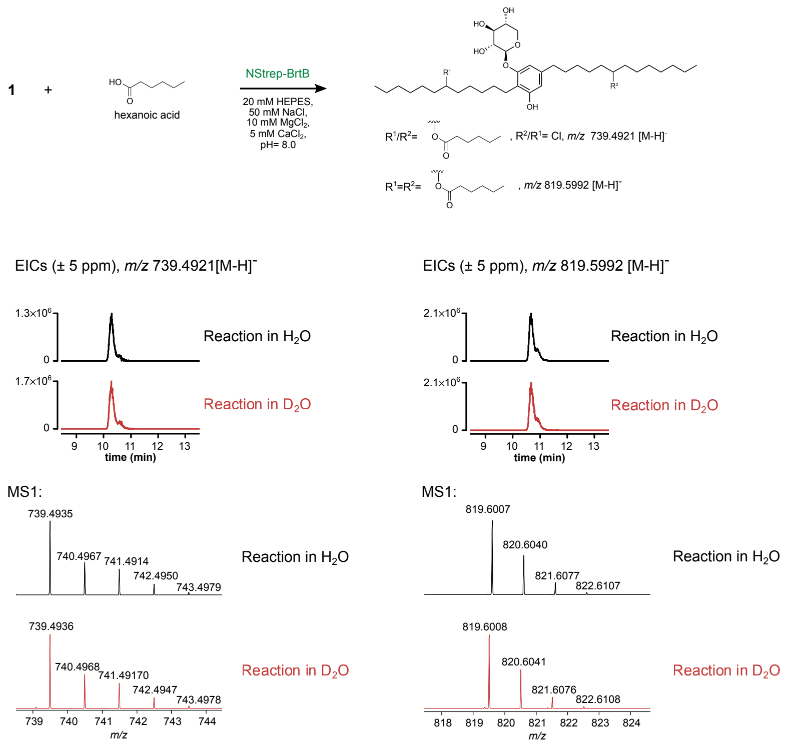

Supplementary Figure 10 – LC-HRESIMS analysis of BrtB assays (16 h) in D_2O-resuspended reaction buffer.

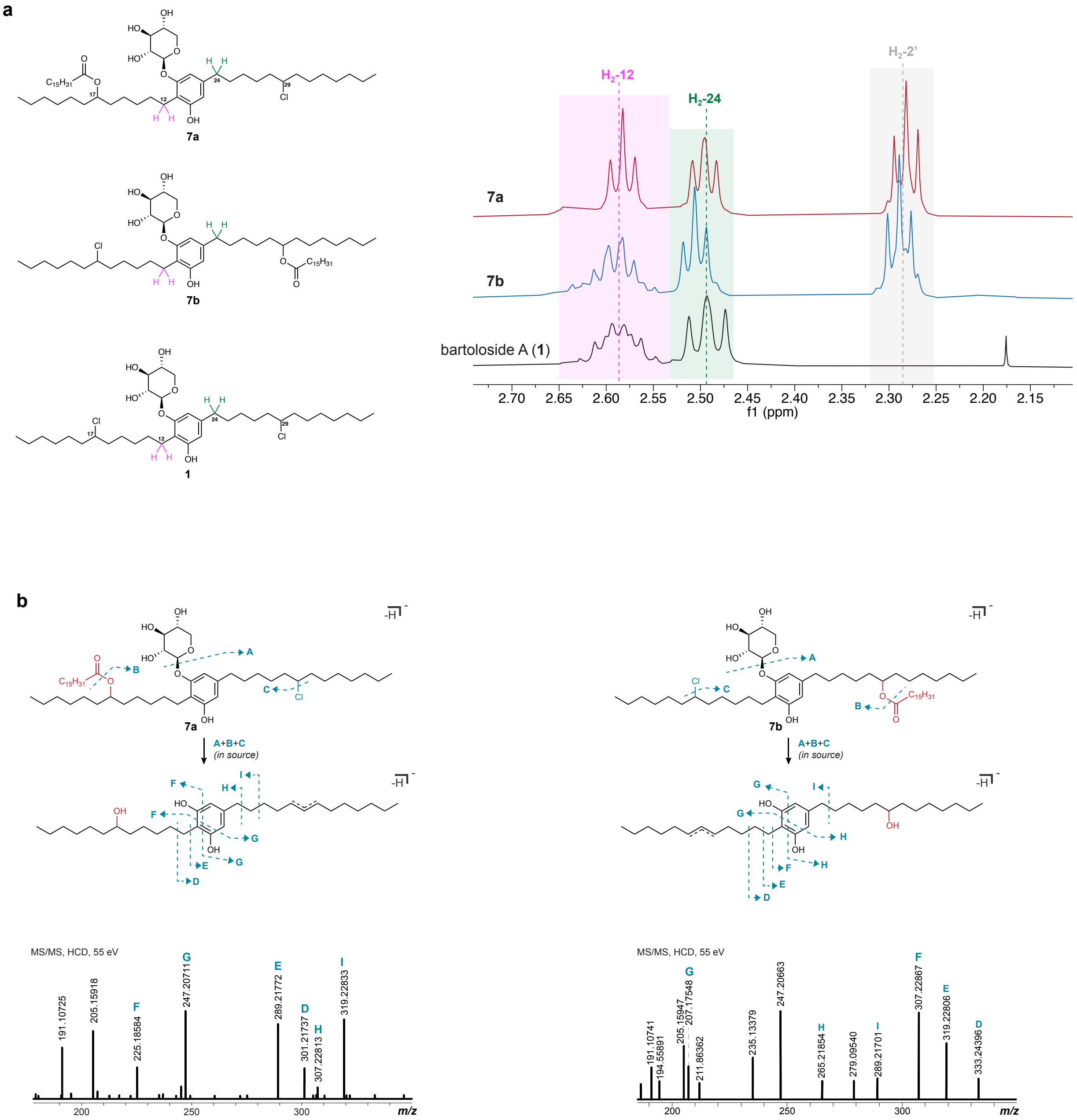

Supplementary Figure 11 – ^{1}H NMR- and HRESIMS/MS based assignment of the esterified alkyl chain in **7a** and **7b**. (a) Changes in multiplicity for the H_2-12 protons in **7a** when compared to **1** and **7b**. (b) Annotated LC-HRESIMS/MS spectra from purified **7a** and **7b**.

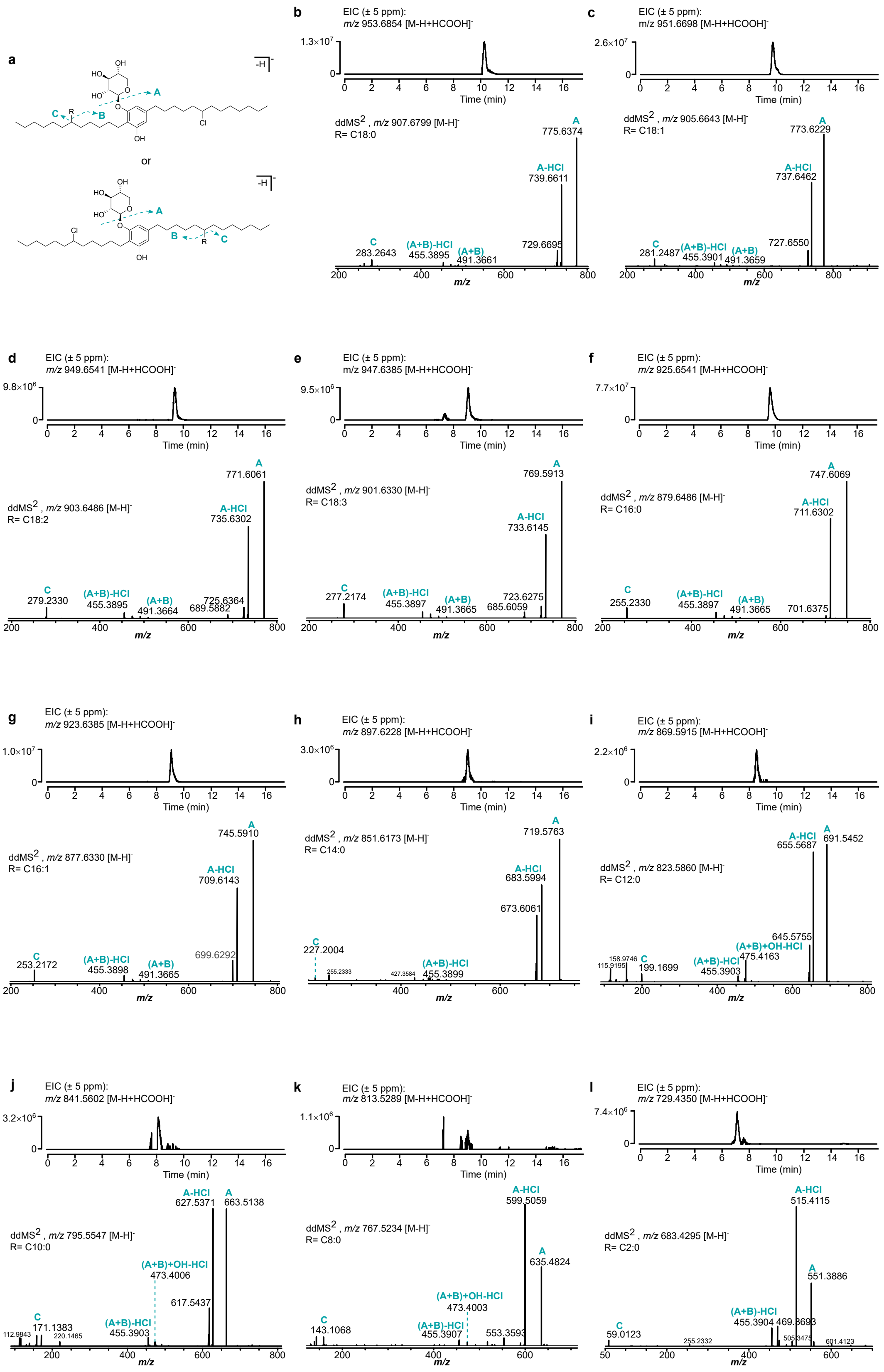

Supplementary Figure 12 – Detection and HRESIMS/MS based structural assignment of natural fatty acid bartoloside monoesters produced by *S. salina* LEGE 06099. a) Reference for the structures of fatty acid bartoloside monoesters and for their annotated MS/MS data. b-l) Extracted Ion Chromatograms (EICs, top) for the [M-H+HCOOH]- ion and MS/MS spectra for the [M-H]- ion of each of the detected esters.

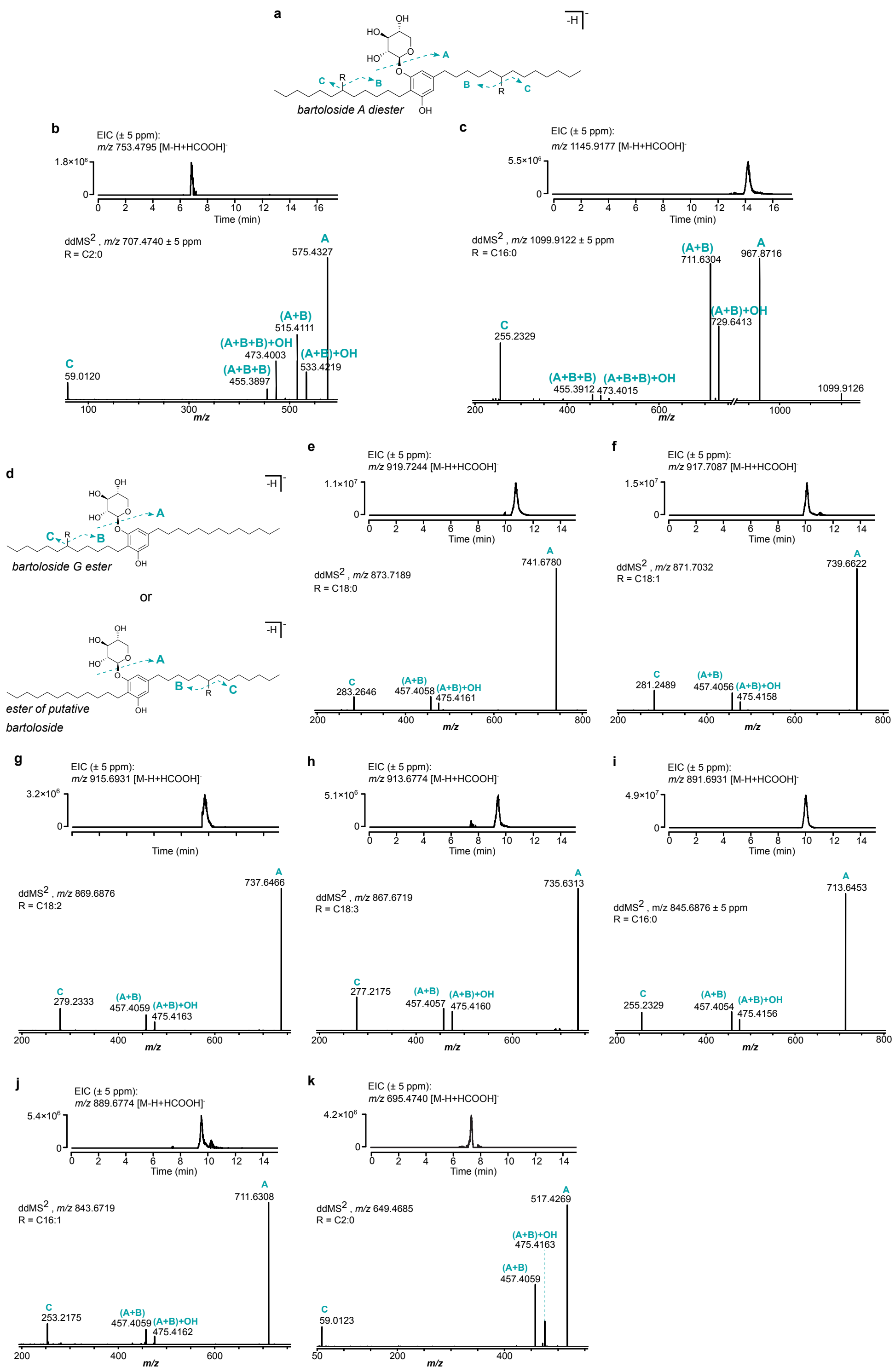

Supplementary Figure 13 – Detection and HRESIMS/MS based structural assignment of natural fatty acid bartoloside esters produced by *S. salina* LEGE 06099. a) Reference for the structures of fatty acid bartoloside A diesters and for their annotated MS/MS data. b-c) Extracted Ion Chromatograms (EICs, top) for the [M-H+HCOOH]$^-$ ion and MS/MS spectra for the [M-H]$^-$ species of each of the detected bartoloside A diesters. d) Reference for the structures of bartoloside G esters or, potentially, esters of a hypothetical structural isomer of bartoloside G that, for example, contains a single halogenation in the opposite chain of the halogenated chain in bartoloside G. e-k) Extracted Ion Chromatograms (EICs, top) and MS/MS spectra for the [M-H]$^-$ ion of each of the detected bartoloside G (or hypothetical structural isomer).

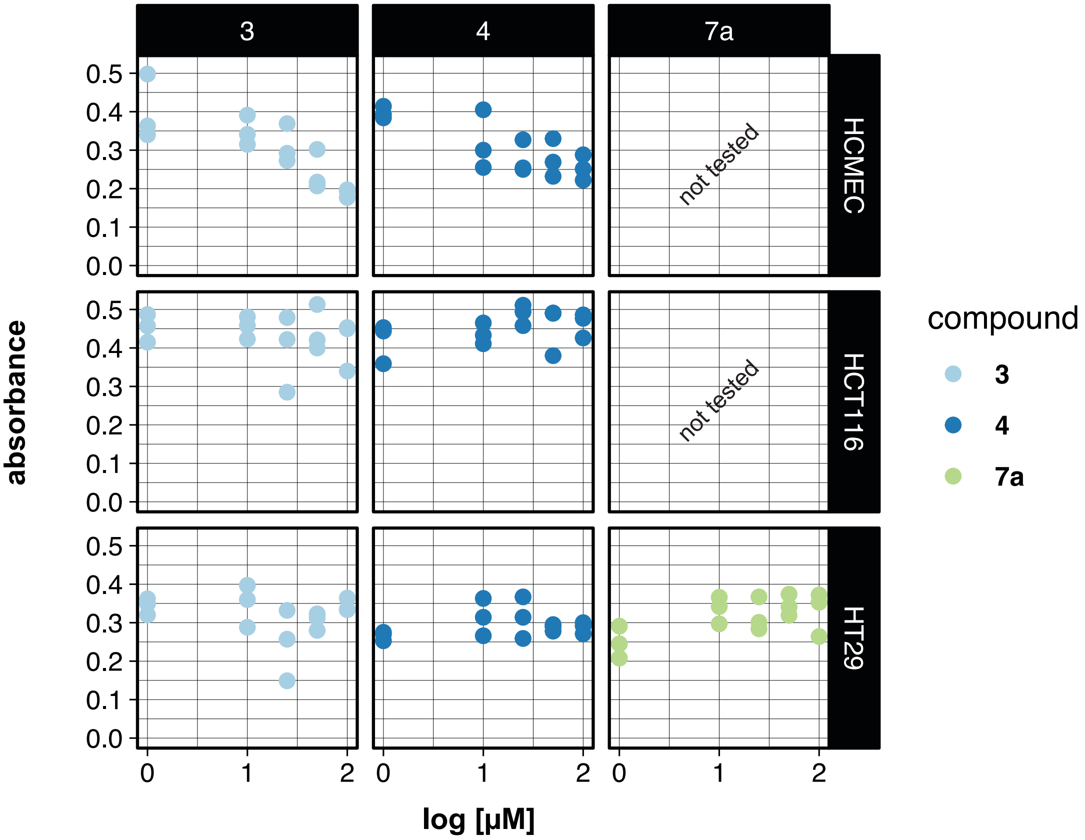

Supplementary Figure 14 – Cytotoxicity assays against immortalized human cells. Each plot depicts cell viability (MTT assay) following a 48 h exposure to selected bartoloside esters (n = 3 biologically independent replicates).

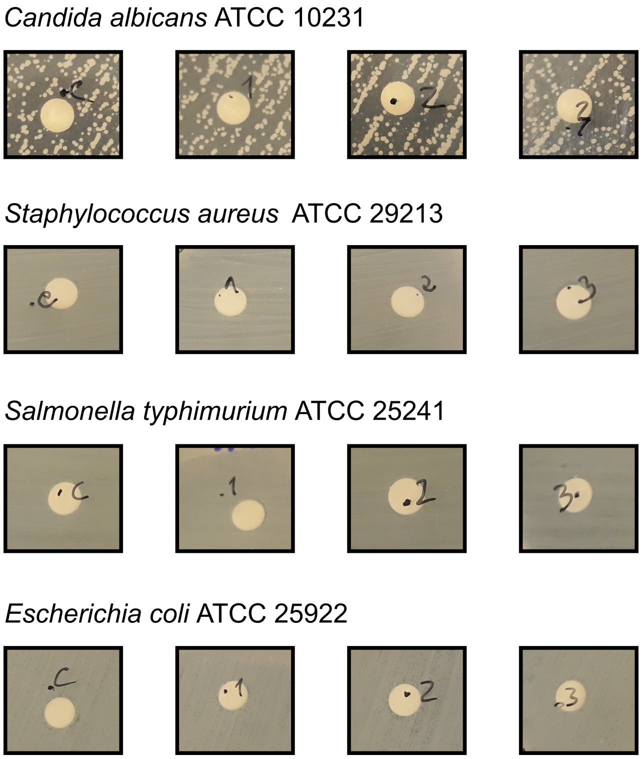

Supplementary Figure 15 – Antimicrobial assays. Photographs of agar disk diffusion assays after 24 h exposure of target bacterial cells to disks containing 15 µL of a 0.5 mg mL^{-1} solution (in DMSO) of selected bartoloside esters. C – negative control (DMSO); 1 – compound **3**; 2 – compound **4**; 3 – compound **7a**.

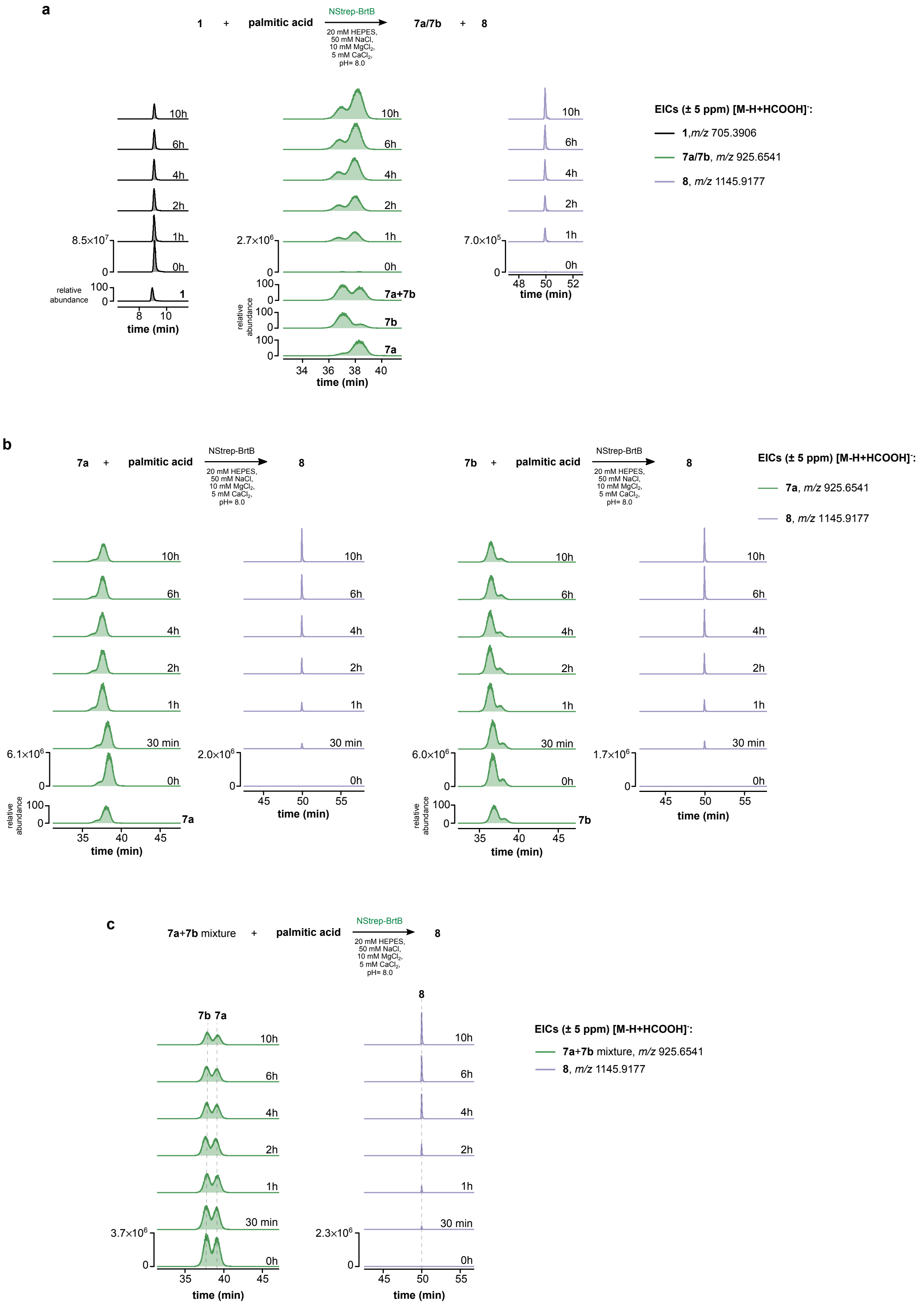

Supplementary Figure 16 – Selectivity of esterification reactions between **1** and palmitic acid. LC-HRESIMS analysis of reactions of BrtB and palmitic acid with **1** (a), **7a** (b, left), **7b** (b, right) and an equimolar mixture of **7a** and **7b** (c).

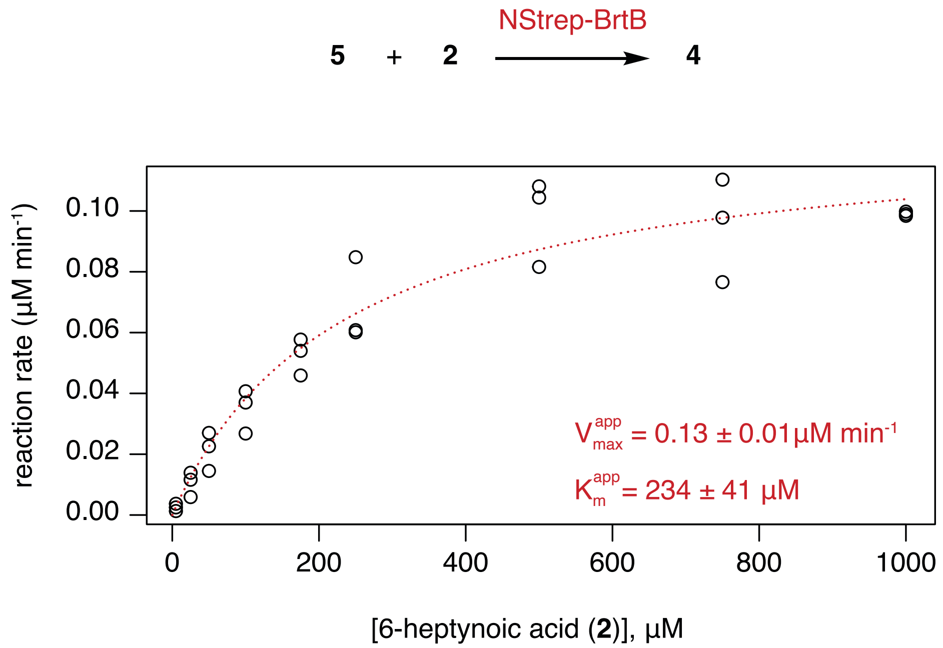

Supplementary Figure 17 –Michaelis-Menten kinetic data for NStrep-BrtB. Apparent K_m and V_{max} were calculated from the non-linear least squares fit of the Michaelis-Menten equation to the kinetic data obtained (± standard error, n=3 independent assays) for different concentrations of 6-heptynoic acid (**2**) and constant bartoloside G (**5**) concentration (100 µM). Source data are provided as a Source Data file.

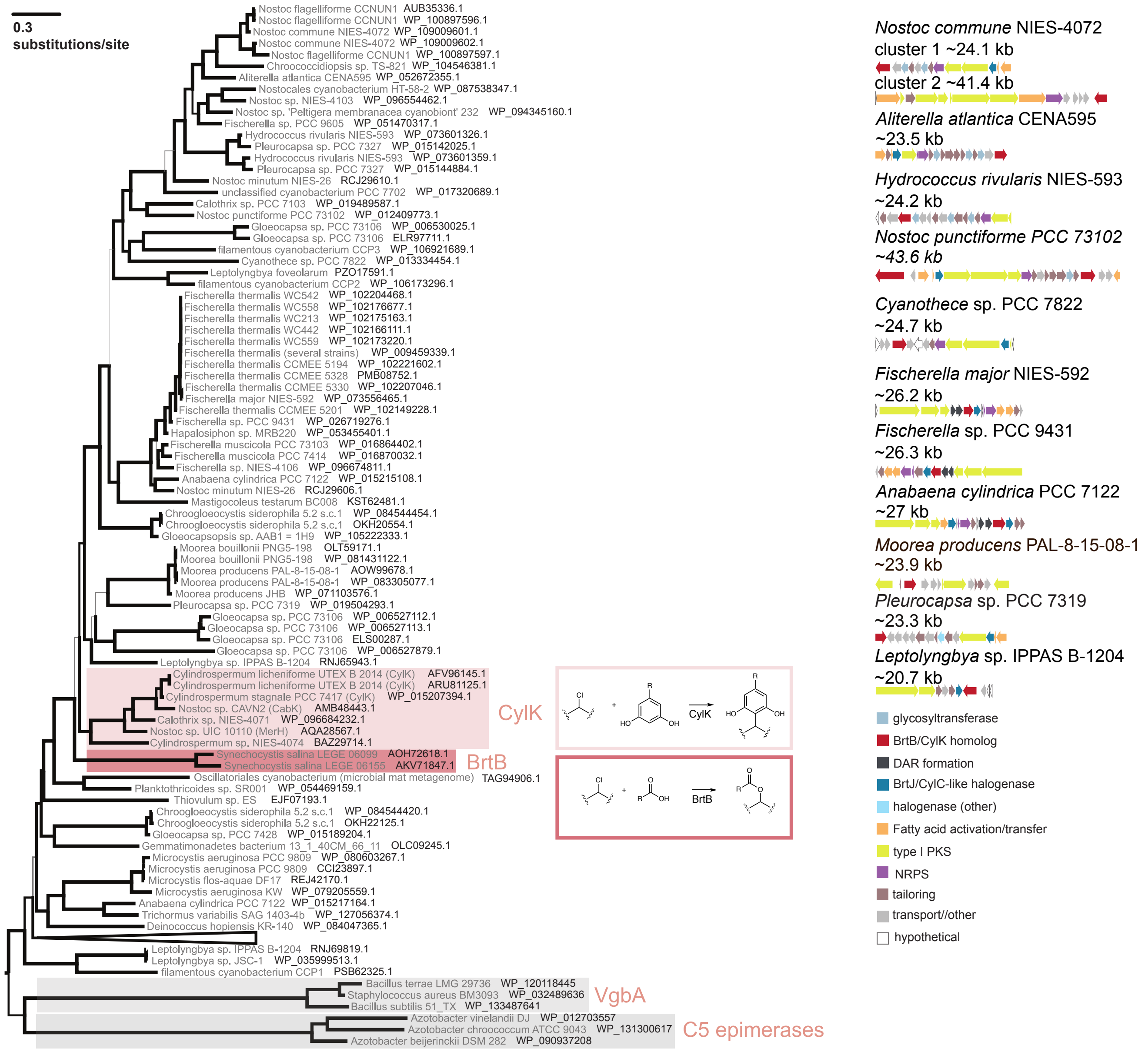

Supplementary Figure 18 – Phylogeny of BrtB homologs and diversity of their associated biosynthetic gene clusters. An approximately-maximum-likeihood tree was built from an alignment of BrtB with its closest homologs, as well as with the more distantly related virginiamycin lyase (VgbA) and alginate C5-epimerases (left panel). The thickness of the tree branches corresponds to bootstrap support (thicker branches have at least 60% support). Right panel - selected biosynthetic gene clusters that contain BrtB homologs. Annotation of the genomic context of *brtB* homologs was carried out using BlastP.

Supplementary Note 2 – Protein and DNA sequences

Protein Sequence (NStrep-BrtB, 91.4 kDa)

MASWSHPQFEKGADDDDKVPDPTSVDMANPFDVALTNLNSLYSGIPDVPVLQTYNSLNPYDISLTNLNNLSNFVIEPAVLPTA
AGTYGPQISEIAFALGGGIITGALADLLSTELGIEIENEALITKFLSFWTPGSDYAFGQDYVEFIQGREGFDTIIGYDPGFNS
ALAPVQIDFVLGGPRQDFNFFAPLDEPARYVLGDWRTPYYVDNDIQSEGLNEFMYVSLFTLNPDEFSPDNAENHRIQLYGSLD
NYYFVPTTVVIDGSALDPSLPSIPVPGTEIYYKTGQVFADLIAIVPFQDPNDPNLISAWIDFEGYLPPEQADLSGQTANPLLS
GSPLLQLGYEGIDMGTATALAPDGDIYVAGSTSSLLGPFPRGGSDNFIARYNDDGSLEWIVQFGSTEFDIITDIVSDSAGNVY
ATGWTRGNVETGFGLAPGVQDNWVAKFDANGSQLWVEQFTIDGYLDRSMGIALDEVNGRIHLTGHSNTDGSVADTNDPAIVPN
GNVVTWIAGFDANTGAEDYRNVFEVAQTSNSRGRIDEGFGIDVDDAGNVFSTGWAGENAYDVYLVKSDTDGAVLWTKSFGTGS
NATQSYAWDVASDGTNAYILGWTQGELNTFRDQIPPTDEFAGNATAPLTPQNAYQGGTFDAFVAAYDTDGTELWTWHVGGTGD
DGTFFGKIVAAGDFVYATGYTDGFIGTLGGSNAGDYDAWIGKFDKLTGQTAWIQQIGSTKLDYATGISVDGDDIFVTGFTEGS
LGGLNGGASDAWVAKLNQDGALEAFNVSAPIAPPAPFAPPAPLTPPAPPAPDLSSLQSLGSTPQPTYPALGDSYGTNPFGTQP
TQPTYDLSSMYQNAYYGGFGGYSGYGY

DNA sequence (NStrep-*brtB*)

ATGGCAAGCTGGAGCCACCCGCAGTTCGAAAAGGGTGCAGATGACGACGACAAGGTACCGGATCCGACGTCGGTCGACATGGC
CAATCCCTTCGACGTCGCTTTAACTAATCTTAACAGTCTTTACAGCGGTATCCCTGATGTTCCCGTTCTTCAGACGTACAACT
CTCTTAACCCCTACGACATTTCTTTAACTAATCTTAACAATCTTAGCAACTTCGTCATTGAGCCAGCCGTGCTCCCTACAGCG
GCAGGTACCTACGGCCCTCAAATCAGTGAAATTGCTTTTGCGTTGGGGGGTGGGATCATAACAGGGGCACTAGCTGATCTGCT
ATCCACTGAGTTGGGGATCGAGATCGAGAACGAAGCTCTGATAACTAAGTTCCTTTCCTTCTGGACTCCCGGTTCCGATTACG
CCTTCGGTCAGGATTATGTTGAATTTATTCAAGGTCGGGAAGGCTTTGACACCATCATCGGTTACGACCCCGGATTCAACAGT
GCATTAGCTCCCGTACAAATTGACTTTGTCCTTGGCGGCCCCAGGCAGGACTTTAACTTCTTTGCCCCCTTAGATGAGCCAGC
ACGCTACGTTTTGGGTGATTGGCGGACCCCCTATTATGTGGATAACGATATCCAGAGTGAGGGGCTGAACGAGTTTATGTATG
TTTCGCTCTTCACACTTAACCCGGATGAATTTTCGCCAGACAATGCAGAAAATCATCGTATCCAGCTCTACGGCAGTCTGGAC
AATTACTACTTTGTACCAACGACAGTTGTAATTGATGGAAGCGCCCTAGATCCAAGTCTTCCATCAATTCCCGTCCCAGGCAC
AGAAATTTACTATAAAACCGGGCAGGTTTTTGCAGATCTGATTGCCATTGTGCCTTTCCAGGATCCTAACGACCCAAATCTTA
TTTCCGCCTGGATTGATTTTGAGGGCTATCTGCCCCCGGAACAGGCCGACCTGAGTGGCCAGACCGCGAACCCCTTGCTCTCC
GGTTCTCCCCTCCTTCAATTGGGCTACGAAGGCATCGATATGGGTACGGCAACGGCCCTCGCCCCGGATGGTGATATCTACGT
CGCTGGCTCCACTAGCAGTCTTTTGGGACCTTTTCCCCGTGGAGGTTCAGACAACTTCATCGCCCGCTACAACGACGATGGTA
GCTTGGAATGGATTGTTCAATTTGGTAGCACTGAGTTCGACATAATCACCGACATTGTCAGCGACAGCGCTGGTAACGTTTAT
GCCACCGGTTGGACCCGGGGCAATGTGGAGACGGGTTTTGGCCTGGCCCCAGGTGTTCAAGACAATTGGGTAGCCAAATTTGA
TGCCAATGGCAGCCAACTCTGGGTAGAGCAATTTACCATTGATGGCTACCTCGACCGTTCCATGGGCATTGCCCTCGATGAAG
TCAATGGGAGAATTCACCTGACAGGGCACTCCAACACGGATGGCTCCGTTGCGGATACAAACGACCCCGCCATTGTCCCCAAT
GGTAATGTGGTCACCTGGATTGCTGGTTTTGATGCTAACACTGGAGCAGAAGACTATCGCAATGTCTTTGAAGTCGCCCAAAC
CTCTAACAGCCGGGGACGCATTGATGAAGGTTTTGGCATAGATGTTGACGATGCTGGCAATGTGTTCTCCACCGGTTGGGCCG
GAGAAAACGCCTACGACGTCTATCTGGTTAAATCCGATACGGATGGCGCTGTACTCTGGACCAAGAGCTTCGGAACTGGGTCT
AATGCCACCCAAAGTTATGCCTGGGATGTTGCTAGTGATGGCACCAATGCCTATATCCTAGGTTGGACCCAGGGCGAACTCAA
CACATTCCGCGATCAAATTCCTCCCACGGATGAATTTGCGGGTAATGCGACGGCTCCACTTACTCCGCAAAATGCTTACCAAG
GTGGTACCTTCGATGCCTTTGTTGCCGCCTATGACACCGATGGTACTGAACTCTGGACTTGGCATGTAGGTGGAACAGGGGAT
GATGGAACCTTCTTCGGTAAAATTGTCGCTGCTGGGGACTTTGTTTACGCCACAGGCTATACCGATGGCTTTATTGGCACCCT
AGGTGGCAGTAACGCTGGTGACTACGATGCCTGGATCGGTAAGTTTGATAAGTTAACCGGTCAGACTGCATGGATCCAGCAGA
TTGGTAGCACCAAGCTAGACTACGCCACTGGTATTTCCGTTGATGGCGATGACATTTTCGTAACAGGCTTTACGGAAGGTTCC
TTGGGCGGCTTGAACGGTGGTGCTTCCGACGCCTGGGTTGCTAAGTTAAATCAAGATGGCGCTTTAGAAGCTTTTAATGTTTC
TGCCCCTATTGCTCCCCCAGCTCCTTTTGCTCCCCCAGCCCCTCTTACTCCTCCAGCTCCCCCGGCTCCTGATCTCAGTAGCT
TGCAGTCATTGGGTAGCACCCCTCAGCCGACCTACCCGGCCCTTGGGGACAGCTATGGCACTAACCCCTTTGGTACTCAGCCT
ACTCAACCTACCTATGACCTCAGCAGCATGTACCAAAATGCTTACTATGGCGGCTTCGGAGGTTATTCCGGGTACGGCTACTA
G

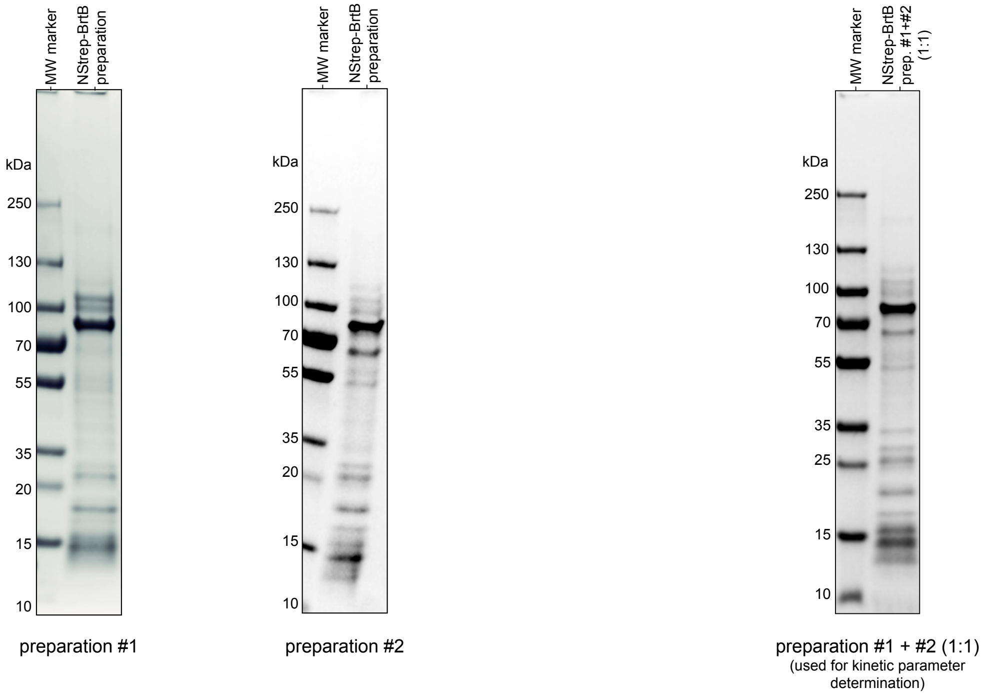

Supplementary Figure 19 – SDS-PAGE analysis of the Strep-NBrtB protein preparations, following purification and concentration. Left: 2 L preparation; Middle: 4 L preparation. For the determination of kinetic parameters, the protein preparations #1 and #2 were pooled in a 1:1 (v/v) ratio (right). Five microliters of each protein sample were loaded into the SDS-PAGE gel. Source data are provided as a Source Data file.

Supplementary Table 1 – NMR Spectroscopic Data (^{1}H 400 MHz, ^{13}C 100 MHz, $CDCl_3$) for bartoloside A-17,29-diyl bis(hept-6-ynoate) (**3**).

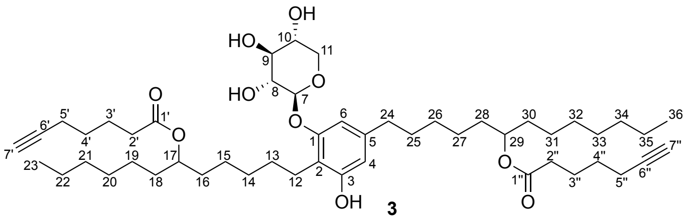

position	δC	type	δH[a]	mult, *J* (Hz)	HMBC[b]	COSY
1	155.8	C				
2	116.3	C				
3	154.6	C-OH				
4	110.4	CH	6.36	d, 1.4	154.6, 116.3, 107.8, 35.6	6.47, 2.48
5	141.9	C				
6	107.8	CH	6.47	d, 1.4	155.8, 116.3, 110.4, 35.6	6.36, 2.48
7	101.4	CH	4.99	d, 5.6	155.8, 74.9, 64.5	3.71, 3.70
8	72.6	CH-OH	3.71	m	101.4, 74.9	4.99, 3.80
9	74.9	CH-OH	3.70	m	72.6, 69.7	4.99, 3.80
10	69.7	CH-OH	3.80	m	74.9	4.10, 3.71, 3.70, 3.45
11	64.5	CH_2	4.10/3.45	dd, 11.9, 4.4/12.0, 8.2	101.4, 74.9, 69.7	3.80, 3.45/4.10, 3.80
12	23.3	CH_2	2.58	t, 7.8	155.8, 154.6, 116.3, 29.3	1.51-1.49, 1.50
13	29.3	CH_2	1.50	m		2.58
14, 20, 26, 32, 33	29.6-29.0	5 × CH_2	1.32-1.28	m	31.9, 31.8, 29.6-29.0, 14.2, 14.1	1.51-1.49, 0.87
15a, 19a, 27a, 31a	25.4-25.1	4 × CH_2	1.51-1.49	m	74.8, 74-6, 31.9, 31.8, 29.6-29.0	4.87, 2.58, 1.32-1.28, 1.27-1.24
15b, 19b, 27b, 31b			1.27-1.24	m	31.9, 31.8, 29.6-29.0	1.51-1.49
16a, 18a, 28a, 30a	34.3-34.1	4 × CH_2	1.51-1.49	m	74.8, 74.6, 31.9, 31.8, 29.6-29.0, 25.4-25.1	4.87, 1.32-1.28, 1.27-1.24
16b, 18b, 28b, 30b			1.27-1.24	m	29.6-29.0, 25.4-25.1	1.51-1.49
17/29	74.8/74.6	2 × CH	4.87	m	174.1, 173.9, 34.3-34.1, 25.4-25.1	1.51-1.49
21/34	31.9/31.8	2 × CH_2	1.27-1.24	m	29.6-29.0, 25.4-25.1, 14.2, 14.1	1.51-1.49, 0.87
22/35	22.7	2 × CH_2	1.27-1.24	m	31.9, 31.8, 29.6-29.0, 25.4-25.1, 14.2, 14.1	1.51-1.49, 0.87
23/36	14.2/14.1	2 × CH_3	0.87	m	31.9, 31.8, 22.7	1.32-1.28, 1.27-1.24
24	35.6	CH_2	2.48	t, 7.5	141.9, 110.4, 107.8, 34.3-34.1, 30.9, 29.6-29.0	6.47, 6.36, 1.55
25	30.9	CH_2	1.55	m	141.9, 29.6-29.0	2.48
1'/ 1"	174.1/173.9	2 × C				
2'/ 2"	34.3/34.3	2 × CH_2	2.32	dt, 7.4, 2.2	174.1, 173.9, 28.0, 24.3	1.73
3'/3"	24.3	2 × CH_2	1.73	m	174.1, 173.9, 34.3, 28.0, 18.3, 18.2	2.32, 1.55
4'/ 4"	28.0	2 × CH_2	1.55	m	84.1, 34.3, 24.3, 18.3, 18.2	2.20, 1.73
5'/ 5"	18.3/18.2	2 × CH_2	2.20	tt, 7.0, 2.7	84.1, 68.8, 28.0, 24.3	1.95, 1.55
6'/ 6"	84.1	2 × C				
7'/ 7"	68.8	2 × CH	1.95	m	84.1, 18.2, 18.3	2.20

[a]from HSQC; [b]from proton to indicated carbon.

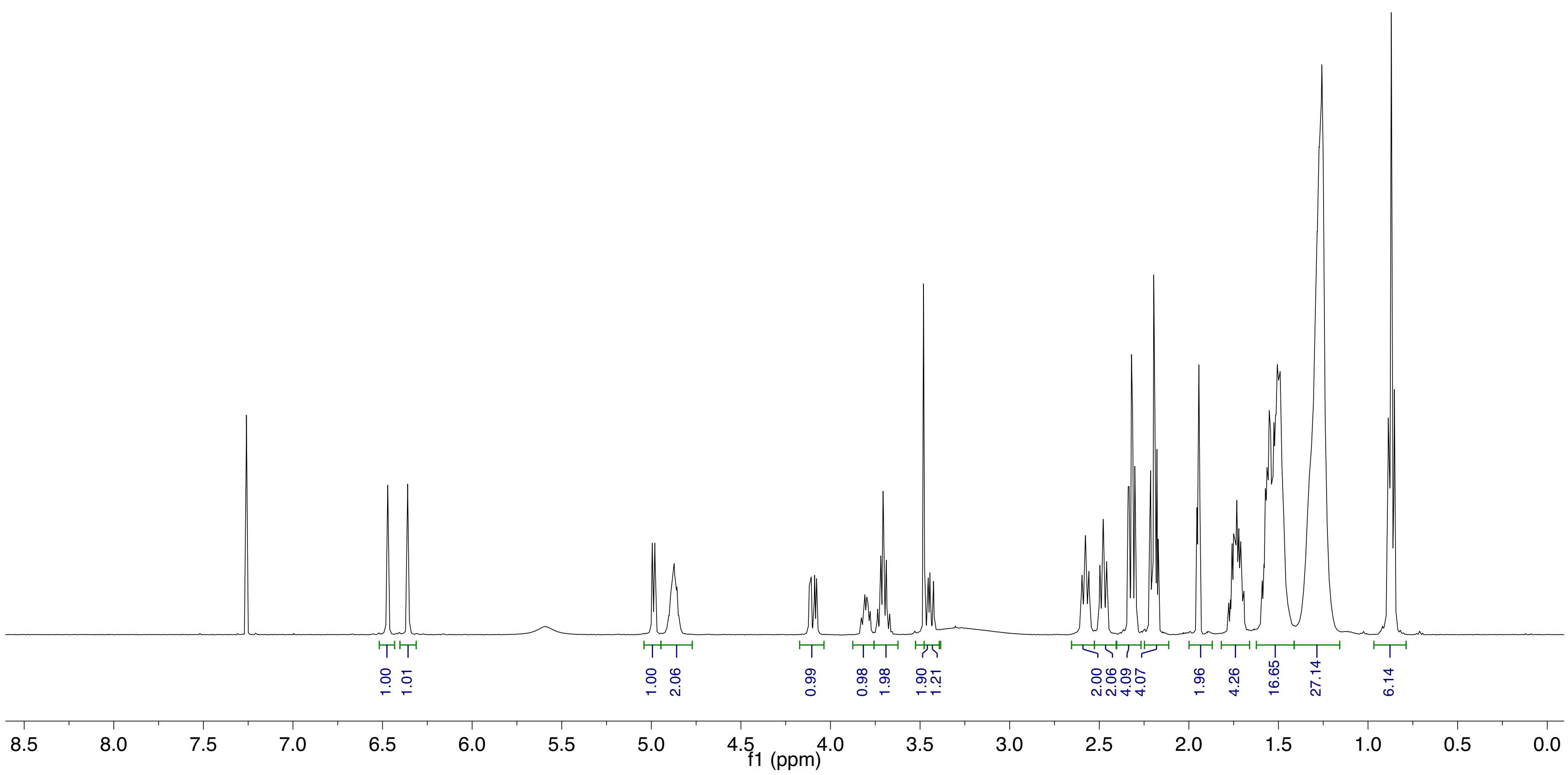

Supplementary Figure 20 – ^{1}H NMR ($CDCl_3$, 400 MHz) spectrum of compound **3**.

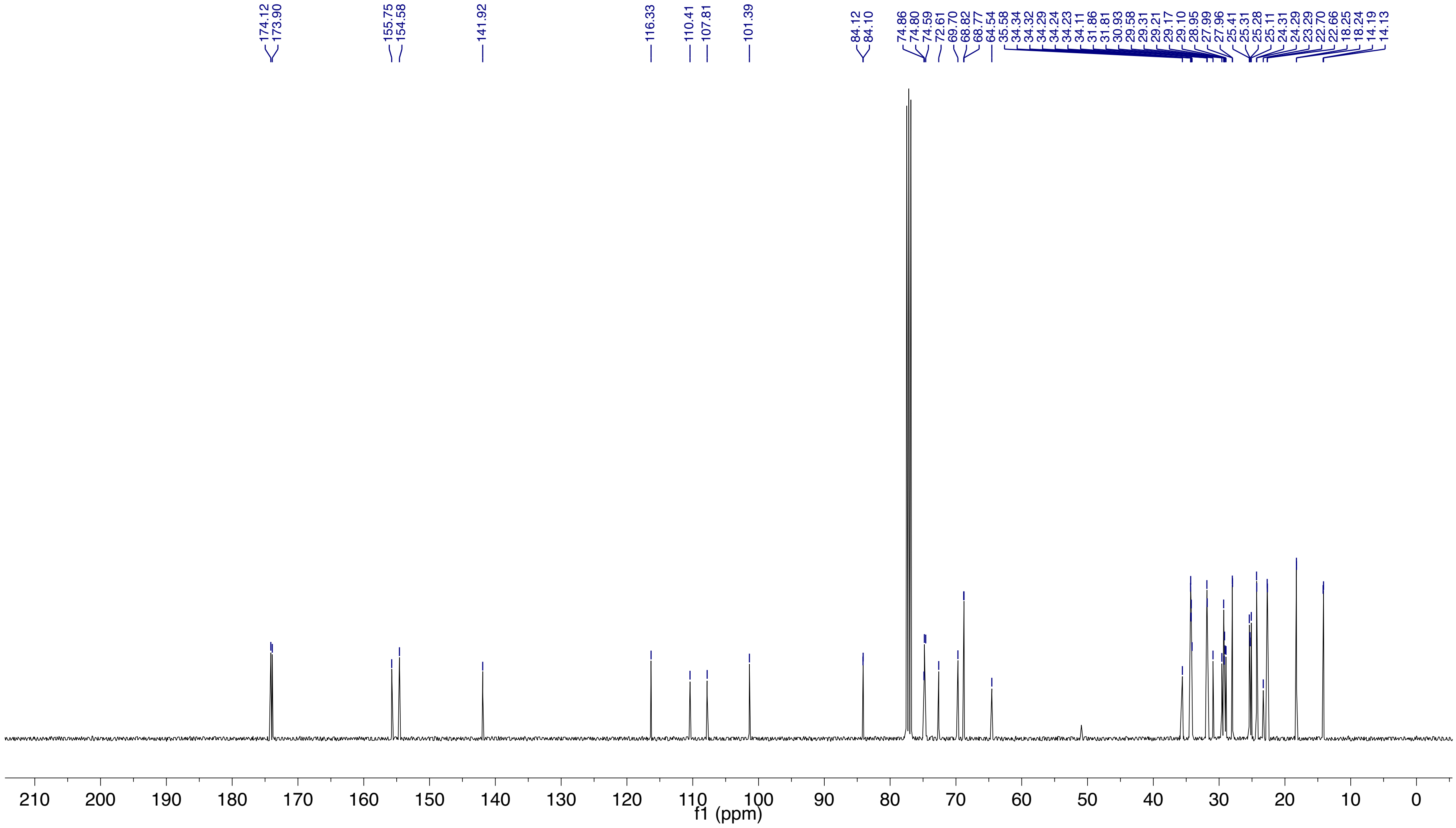

Supplementary Figure 21 – ^{13}C NMR ($CDCl_3$, 100 MHz) spectrum of compound **3**.

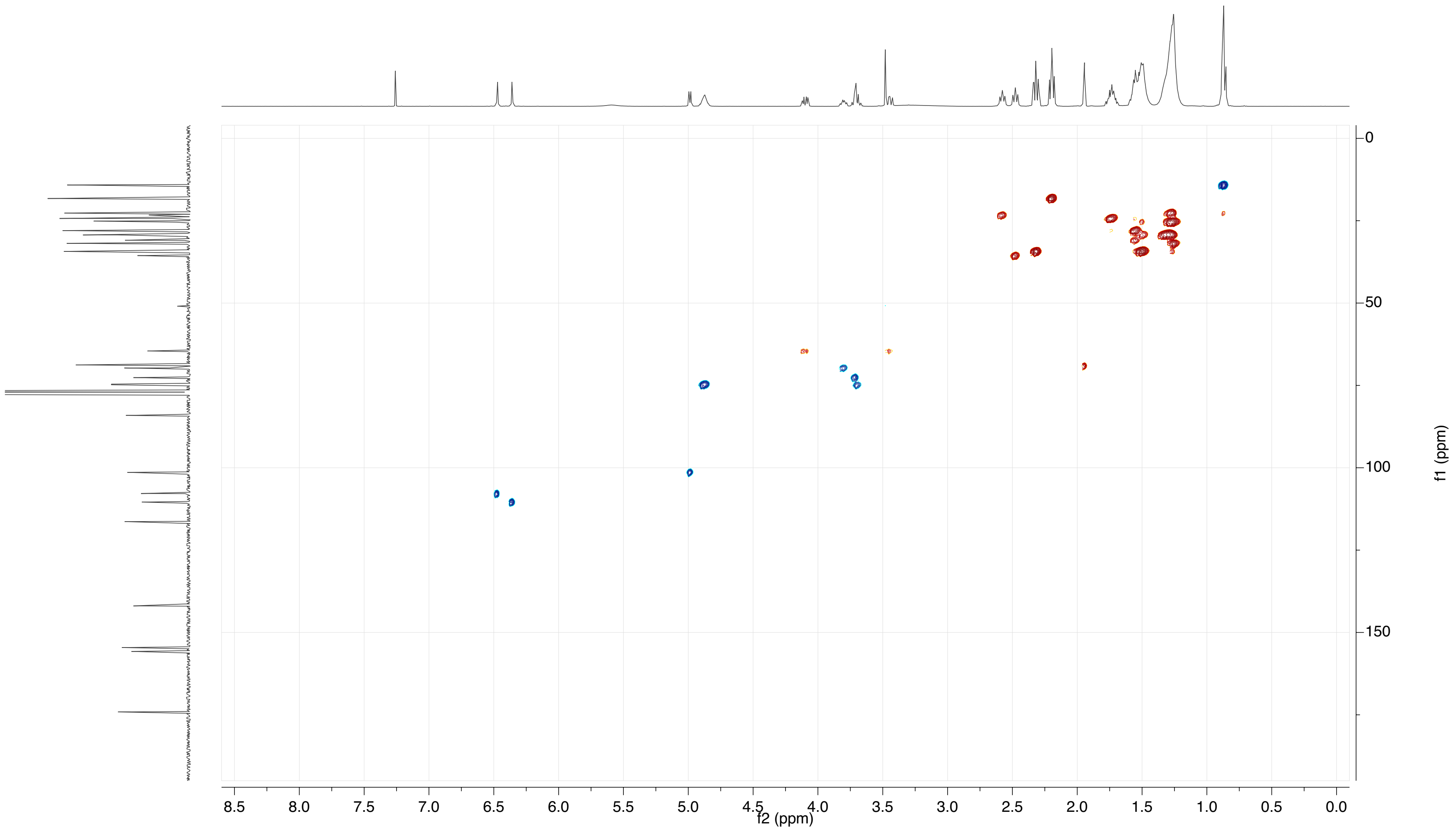

Supplementary Figure 22 – HSQC ($CDCl_3$, 400 MHz) spectrum of compound **3**.

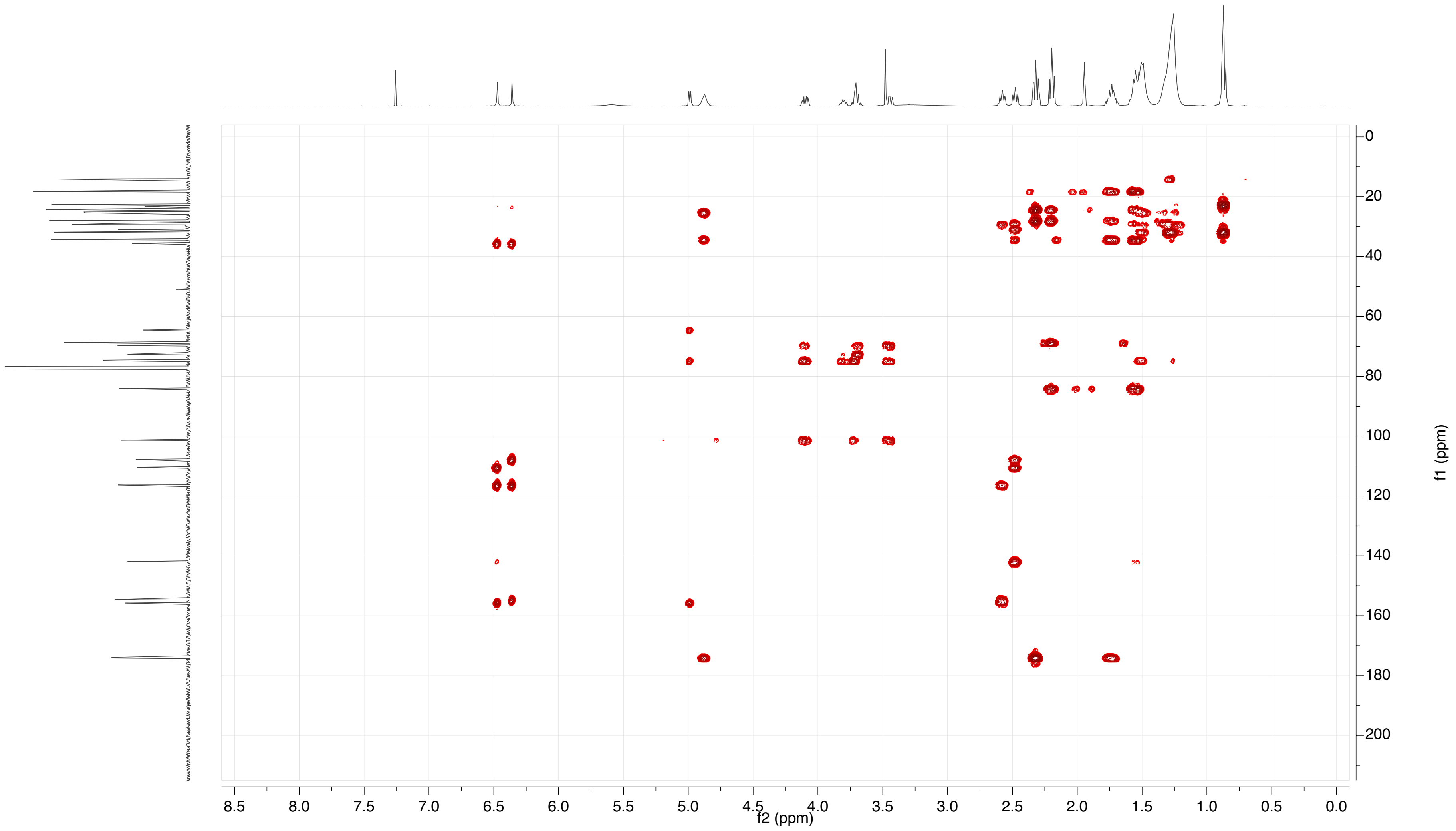

Supplementary Figure 23 – HMBC ($CDCl_3$, 400 MHz) spectrum of compound **3**.

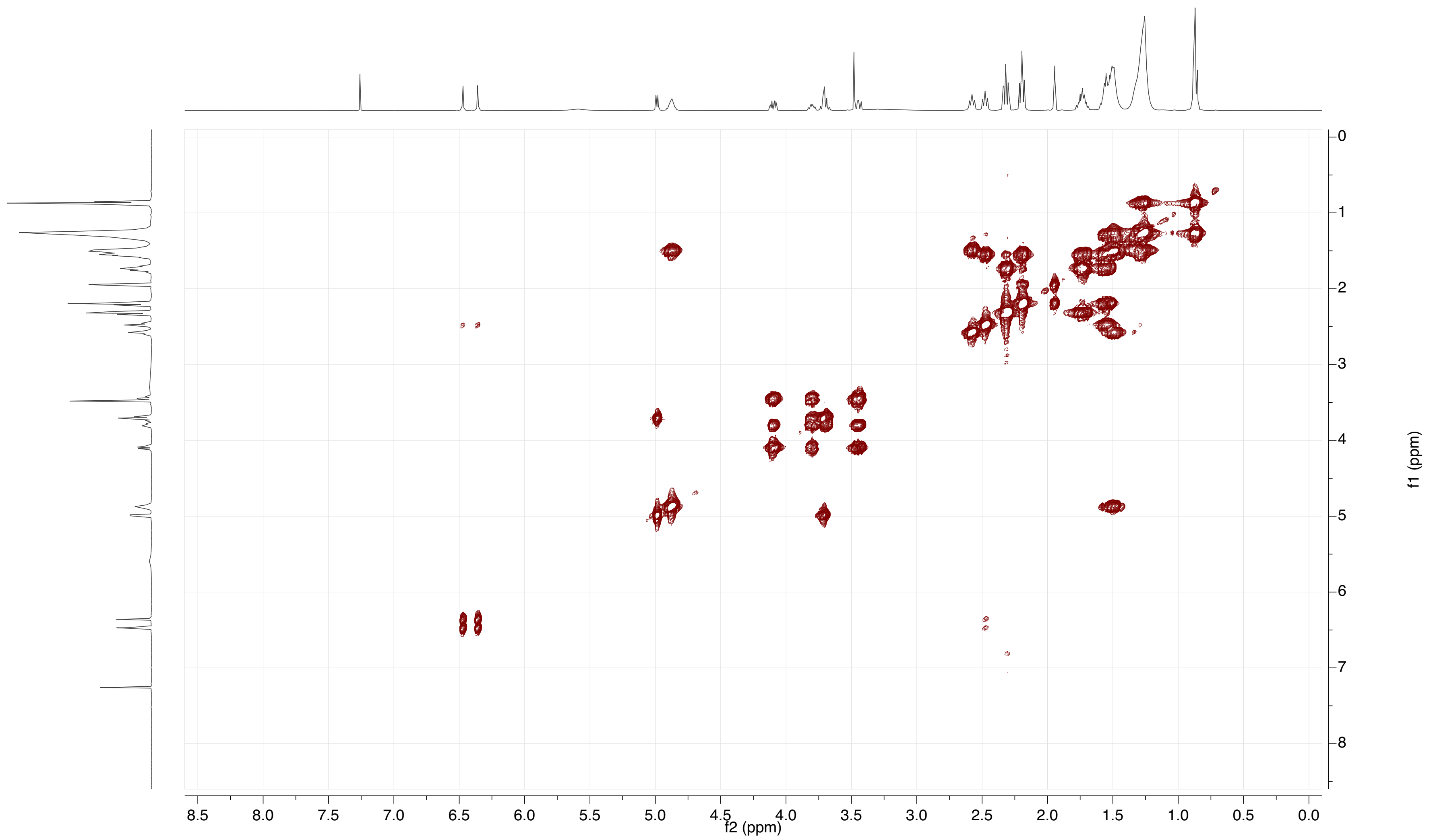

Supplementary Figure 24 – COSY ($CDCl_3$, 400 MHz) spectrum of compound **3**.

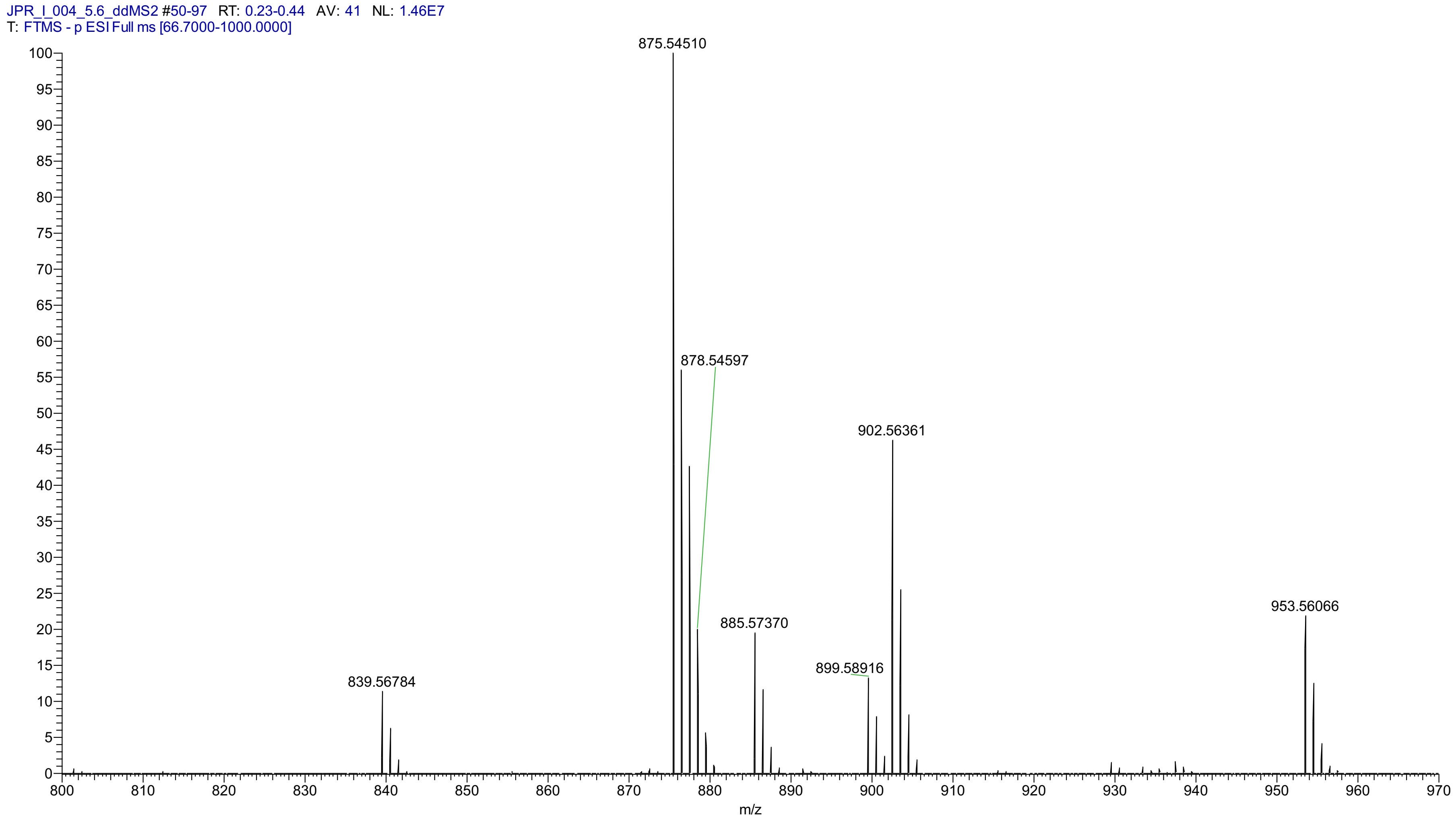

Supplementary Figure 25 – HRESIMS spectrum of compound **3**.

Supplementary Table 2 – NMR Spectroscopic Data (^{1}H 400 MHz, ^{13}C 100 MHz, $CDCl_3$) for bartoloside G-17-yl hept-6-ynoate (**4**)

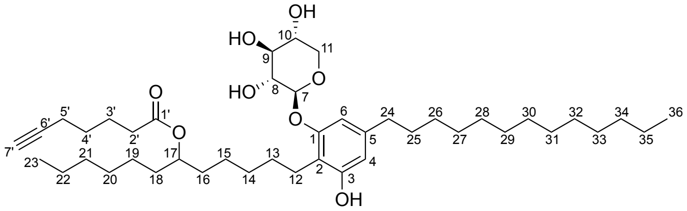

4

**position**	**δC**	**type**	**δH**[a]	**mult, *J* (Hz)**	**HMBC**[b]	**COSY**
1	155.7	C				
2	116.1	C				
3	154.3	C-OH				
4	110.4	CH	6.36	d, 1.5	154.3, 116.1, 108.1, 36.0	6.51, 2,49
5	142.5	C				
6	108.1	CH	6.51	d, 1.5	155.7, 116.1, 110.4, 36.0	6.36, 2.49
7	101.4	CH	5.00	d, 5.7	155.7, 74.9, 64.5	3.72, 3.70
8	72.6	CH-OH	3.72	m		5.00, 3.83
9	74.9	CH-OH	3.70	m		5.00, 3.83
10	69.7	CH-OH	3.83	m		4.12, 3.72, 3.70, 3.47
11	64.5	CH_2	4.12/3.47	dd, 11.9, 4.4/11.9, 8.1	101.4, 74.9/101.4, 74.9, 69.7	3.83, 3.47/4.12, 3.83
12	23.3	CH_2	2.58	t, 7.8	155.7, 154.3, 116.1, 29.1	1.51-1.50, 1.50
13	29.1	CH_2	1.50	m		2.58
14/20, 26-33	29.9-29.0	10 × CH_2	1.27-1.24	m	32.1, 31.8, 29.9-29.0, 22.8, 22.7, 14.3, 14.2	1.51-1.50, 0.87
15a, 19a	25.3/25.2	2 × CH_2	1.51-1.50	m	32.1, 31.8	4.88, 2.58, 1.30-1.28
15b, 19b			1.30-1.28	m	32.1, 31.8, 29.9-29.5	1.51-1.50
16a, 18a	34.3	2 × CH_2	1.51-1.50	m	32.1, 31.8, 25.3, 25.2	4.88, 1.30-1.28, 1.27-1.24
16b, 18b			1.27-1.24	m	29.9-29.0	1.51-1.50
17	74.7	CH	4.88	p, 6.2	174.1, 34.3, 25.3, 25.2	1.51-1.50
21/34	32.1/31.8	2 × CH_2	1.27-1.24	m	29.9-29.0, 22.8, 22.7, 14.3, 14.2	1.51-1.50, 0.87
22/35	22.8/22.7	2 × CH_2	1.27-1.24	m	32.1, 31.8, 29.85-29.51, 14.3, 14.2	1.51-1.50, 0.87
23/36	14.3/14.2	2 × CH_3	0.87	m	32.1, 31.8, 22.8, 22.7	1.30-1.28, 1.27-1.24
24	36.0	CH_2	2.49	t, 7.7	142.5, 110.4, 108.1, 31.4, 29.9-29.0	6.51, 6.36, 1.56
25	31.4	CH_2	1.56	m	29.9-29.0	2.49
1'	174.1	C				
2'	34.4	CH_2	2.32	t, 7.4	174.1, 28.0, 24.3	1.74
3'	24.3	CH_2	1.74	m	174.1, 34.4, 28.0, 18.3	2.32, 1.55
4'	28.0	CH_2	1.55	m	84.1, 34.4, 24.3, 18.3	2.20, 1.74
5'	18.3	CH_2	2.20	td, 7.0, 2.6	84.1, 68.8, 28.0, 24.3	1.95, 1.55
6'	84.1	C				
7'	68.8	CH	1.95	t, 2.7		2.20

[a]from HSQC; [b]from proton to indicated carbon.

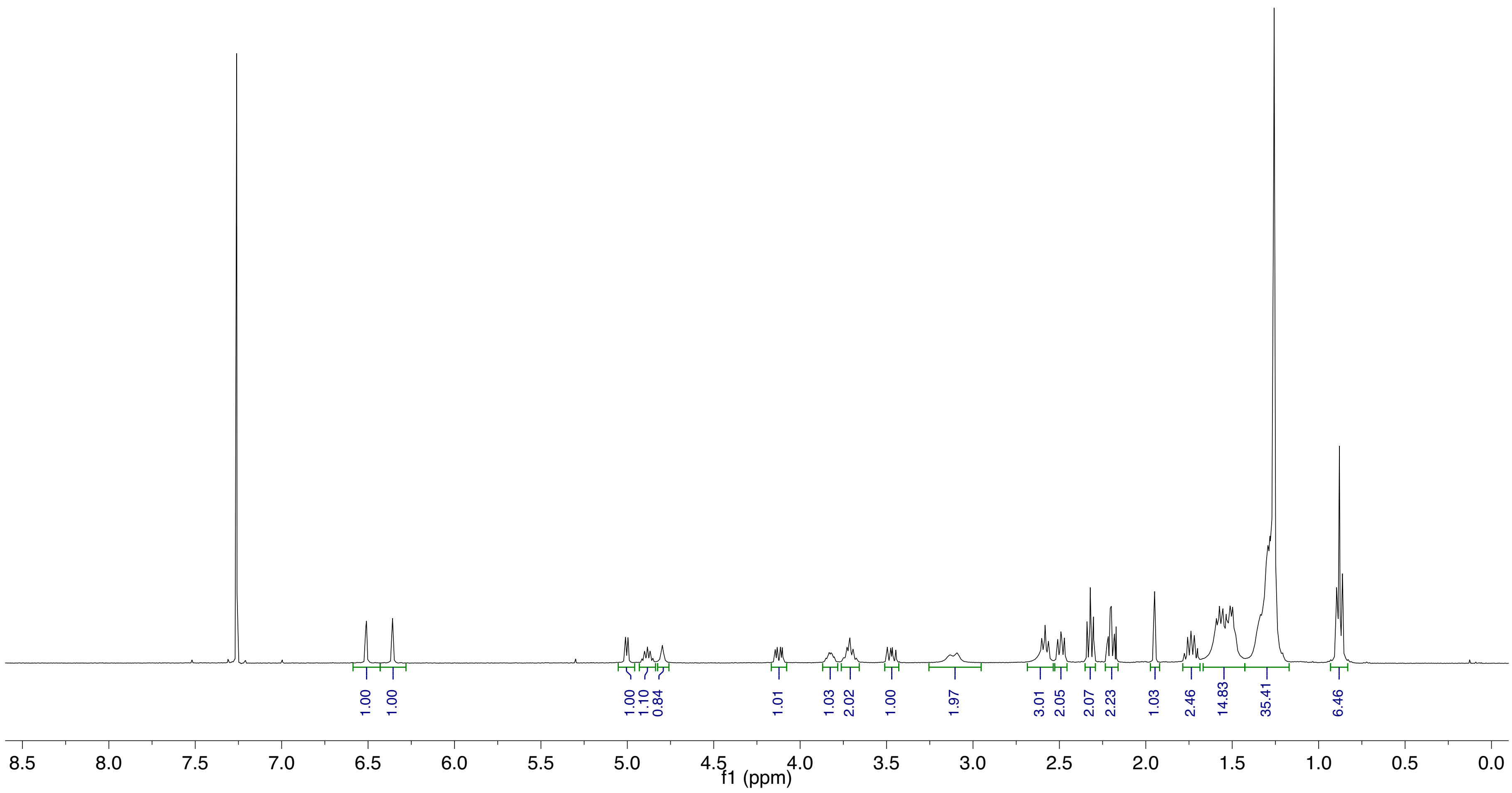

Supplementary Figure 26 – ^{1}H NMR ($CDCl_3$, 400 MHz) spectrum of compound **4**.

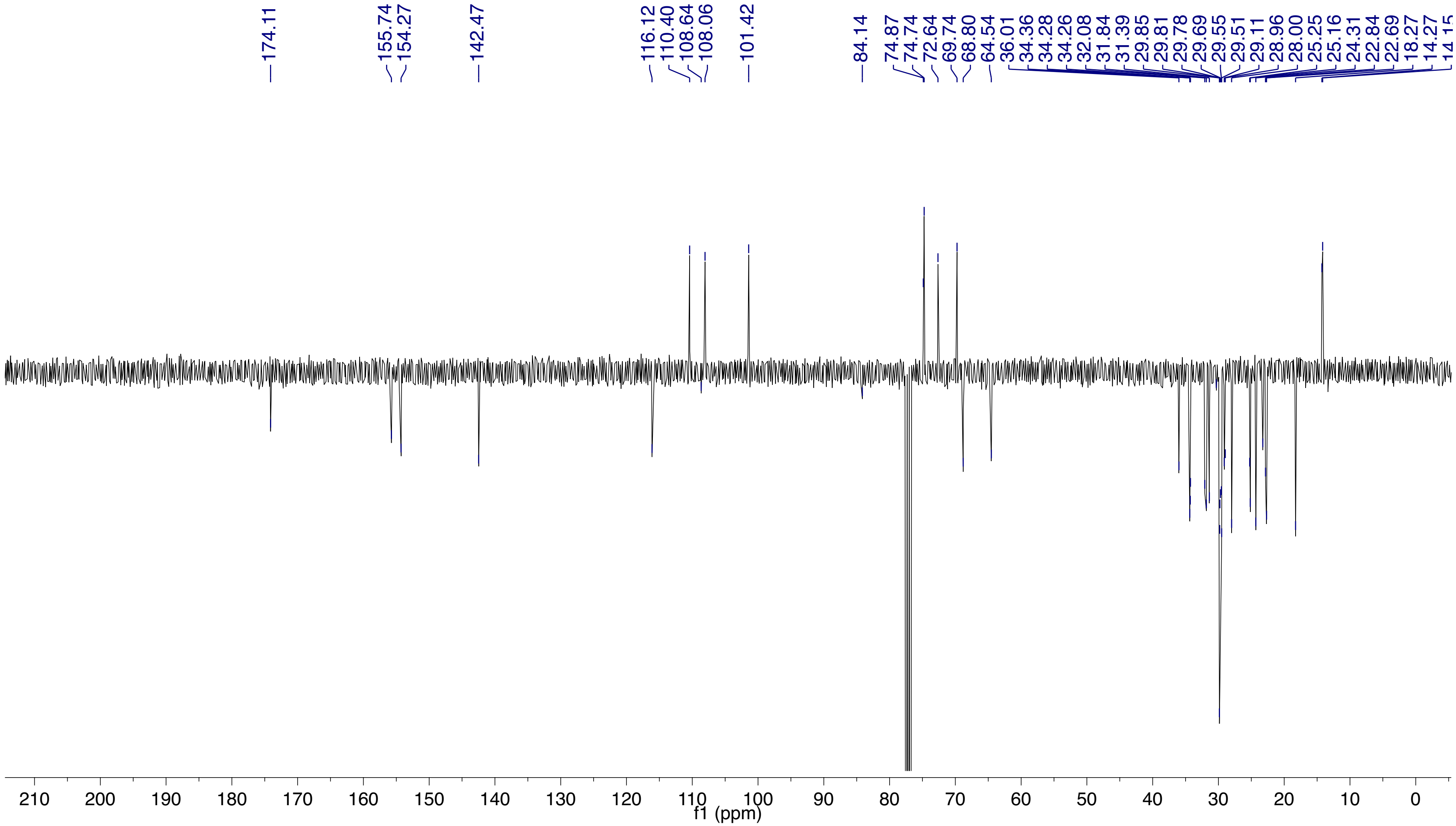

Supplementary Figure 27 – ^{13}C NMR (APT, $CDCl_3$, 100 MHz) spectrum of compound **4**.

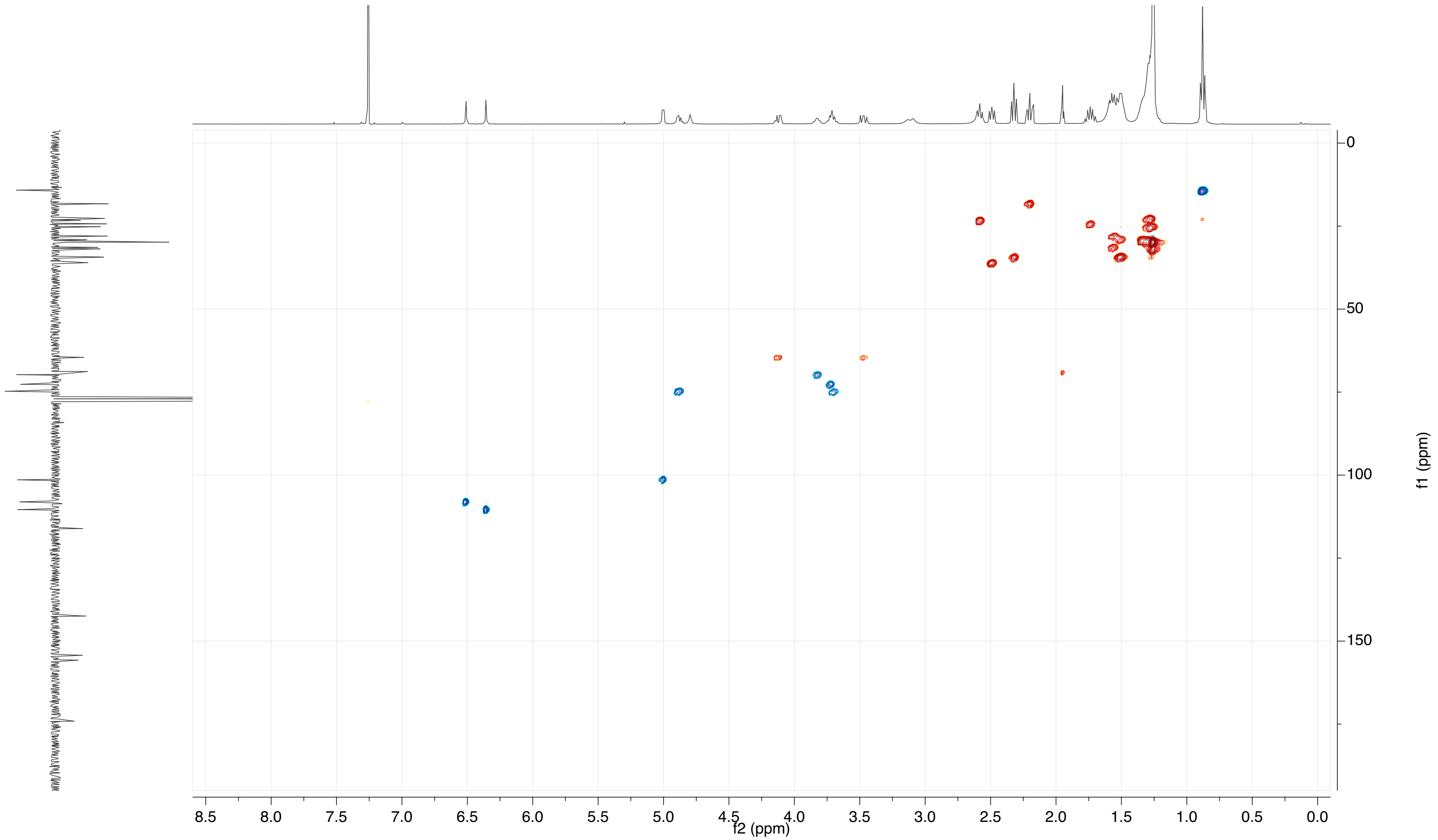

Supplementary Figure 28 – HSQC ($CDCl_3$, 400 MHz) spectrum of compound **4**.

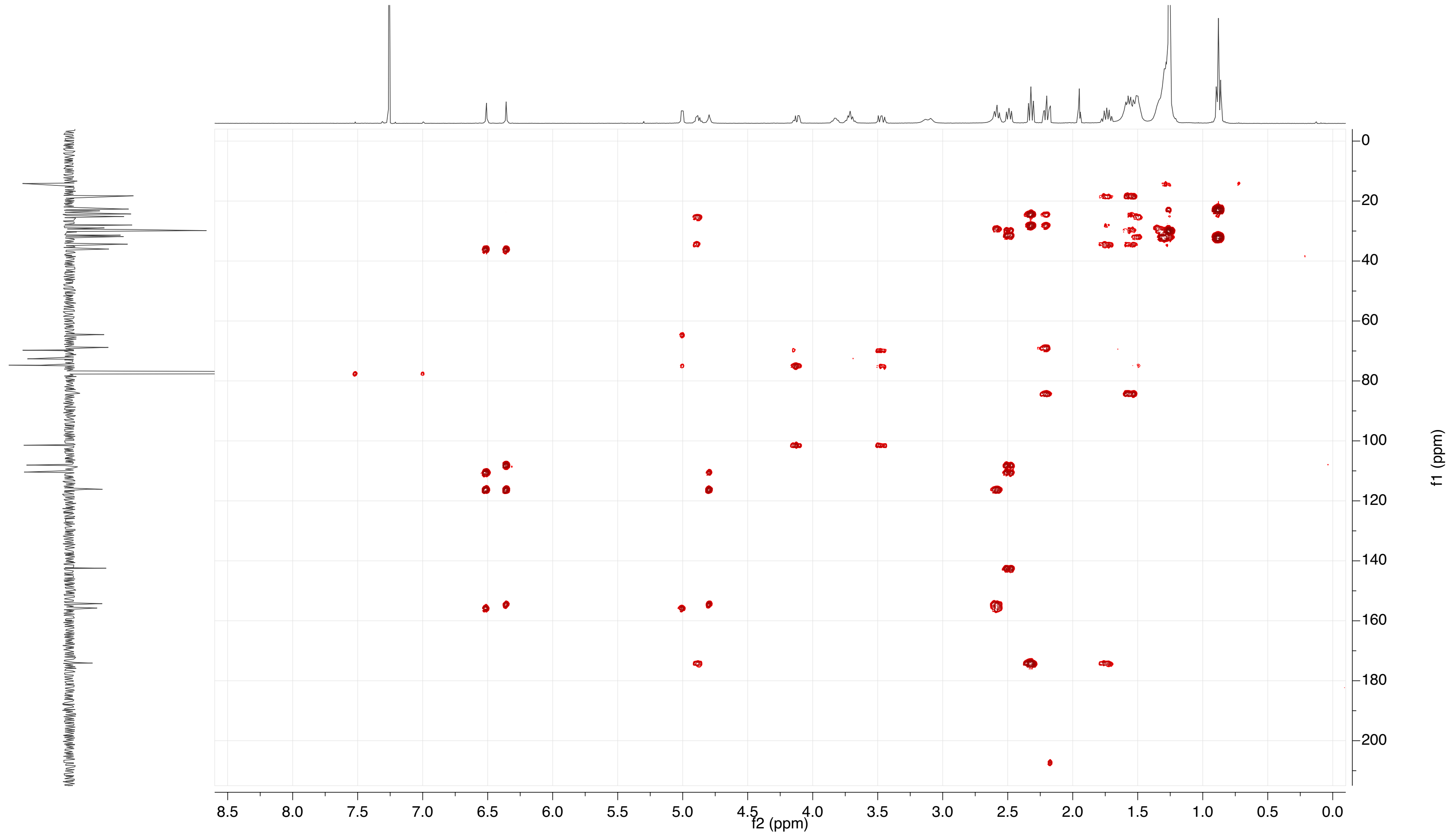

Supplementary Figure 29 – HMBC ($CDCl_3$, 400 MHz) spectrum of compound **4**.

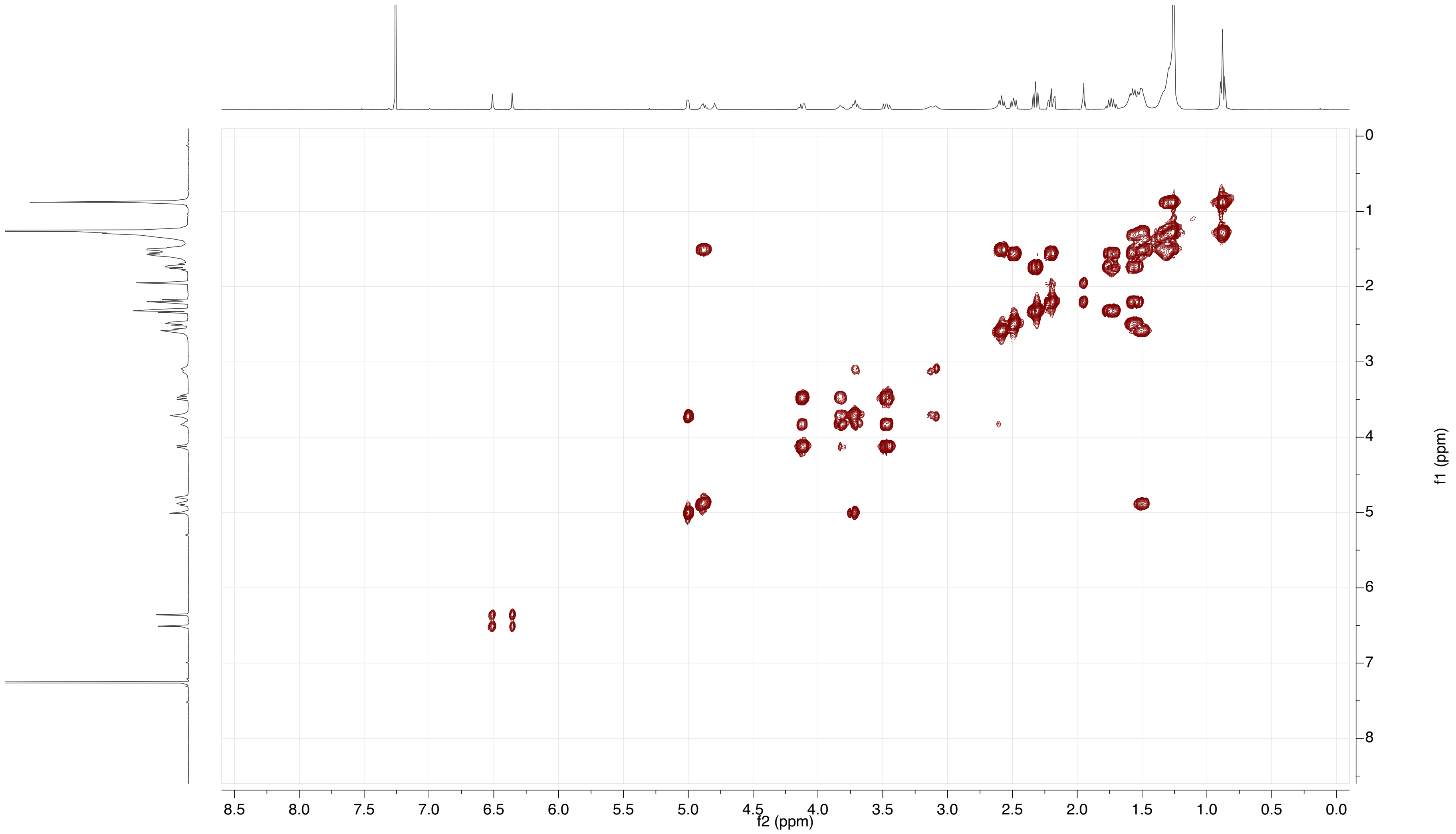

Supplementary Figure 30 – COSY ($CDCl_3$, 400 MHz) spectrum of compound **4**.

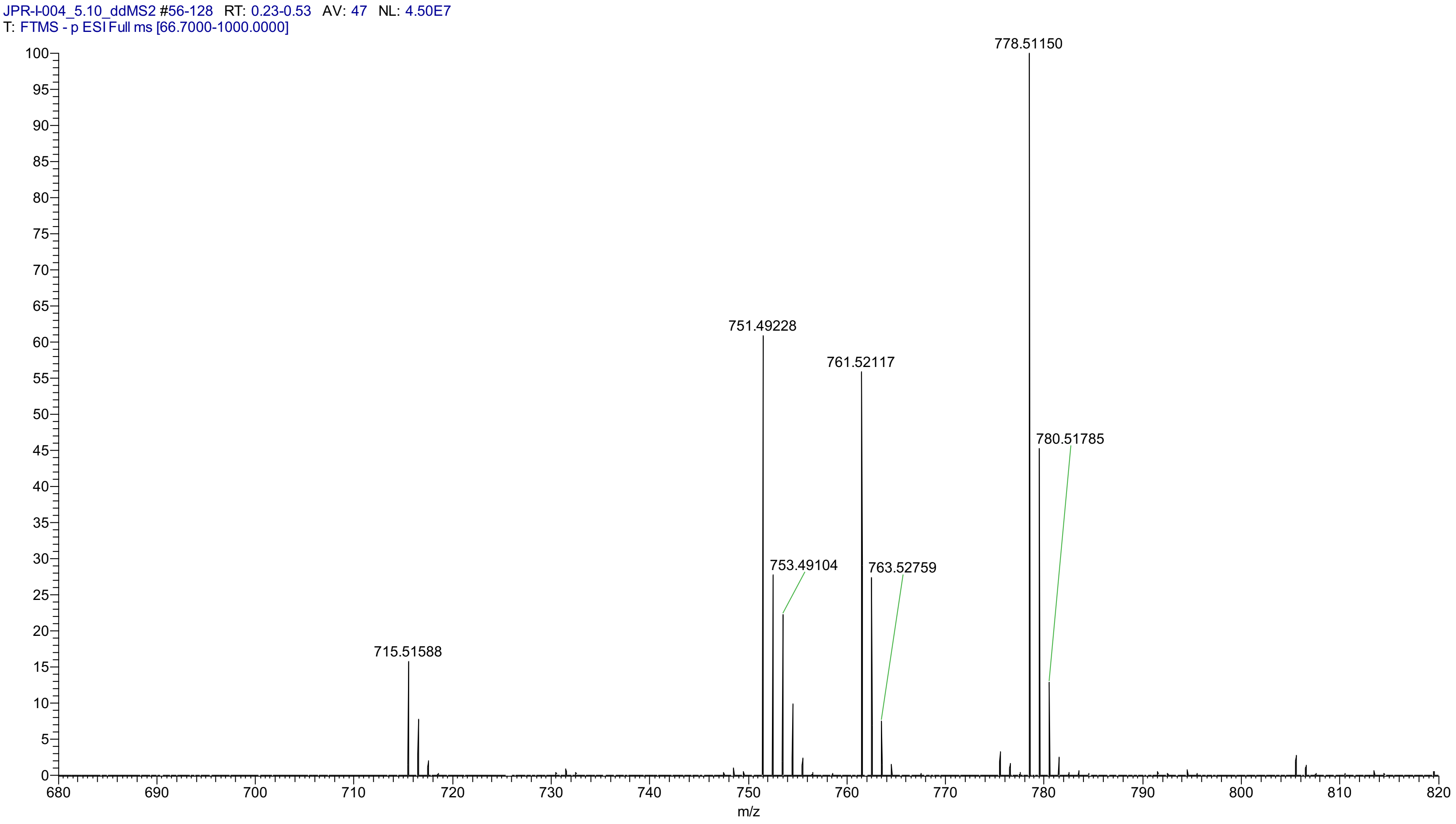

Supplementary Figure 31 – HRESIMS spectrum of compound **4**.

Supplementary Table 3 – NMR Spectroscopic Data (^{1}H 400 MHz, ^{13}C 100 MHz, $CDCl_3$) for bartoloside A-17-yl palmitate (**7a**).

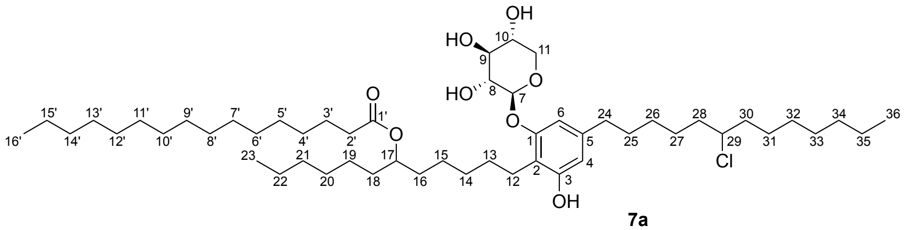

7a

position	δC	type	δH[a]	mult, *J* (Hz)	HMBC[b]	COSY
1	155.8	C				
2	116.2	C				
3	154.3	C-OH				
4	110.4	CH	6.36	s	154.3, 116.2, 108.1, 35.9	6.51
5	142.3	C				
6	108.1	CH	6.51	s	155.8, 116.2, 110.4, 35.9	6.36
7	101.4	CH	5.01	d, 5.7	155.8	3.72, 3.70
8	72.6	CH-OH	3.72	m	101.4, 74.9	5.01, 3.83
9	74.9	CH-OH	3.70	m	72.6, 69.8	5.01, 3.83
10	69.8	CH-OH	3.83	m		4.13, 3.72, 3.70, 3.48
11	64.6	CH_2	4.13/3.48	dd, 11.9, 4.4/dd, 11.9, 8.0	101.4, 74.9/101.4, 69.8	3.83, 3.48/4.13, 3.83
12	23.2	CH_2	2.58	t, 7.7	155.8, 154.3, 116.2, 29.2	1.51, 1.51-1.50
13	29.2	CH_2	1.51	m		2.58
14, 20, 26, 32, 33, 4'-13'	29.9-28.9	15 × CH_2	1.27-1.24	m	29.9-28.9, 22.8-22.7	1.51-1.50, 0.88
15a, 19a	25.2	2 × CH_2	1.61-1.60		29.9-28.9	2.28, 1.30-1.28
15b, 19b			1.30-1.28		32.1-31.9, 29.9-28.9, 22.8-22.7	1.61-1.60, 1.51-1.50
16a, 18a	34.3	2 × CH_2	1.51-1.50	m		4.88, 1.30-1.28, 1.41-1.40
16b, 18b			1.27-1.24	m	29.9-28.9	1.51-1.50
17	74.5	CH	4.88	p, 6.4	174.7, 25.2	1.51-1.50
21, 34, 14'	32.1-31.9	3 × CH_2	1.27-1.24	m	29.9-28.9, 22.8-22.7	1.51-1.50, 0.88
22, 35, 15'	22.8-22.7	3 × CH_2	1.30-1.28	m	32.1-31.9, 29.9-28.9, 22.8-22.7	1.61-1.60, 0.88
23, 36, 16'	14.3-14.2	3 × CH_3	0.88	m	32.1-31.9, 22.8-22.7	1.30-1.28
24	35.9	CH_2	2.5	t, 7.7	142.3, 110.4, 108.1, 31.2	1.58
25	31.2	CH_2	1.58	m		2.5
27a, 31a	26.6	2 × CH_2	1.51-1.50	m		1.7, 1.41-1.40
27b, 31b			1.41-1.40	m		1.51-1.50
28/30	38.7	2 × CH_2	1.70	m	64.5	3.88, 1.51-1.50
29	64.5	CH-Cl	3.88	m	26.6	1.70
1'	174.7	C				
2'	35.0	CH_2	2.28	t, 7.5	174.7, 29.9-28.9, 25.3	1.61-1.60
3'	25.3	CH_2	1.61-1.60	m	29.9-28.9	2.28

[a]from HSQC; [b]from proton to indicated carbon.

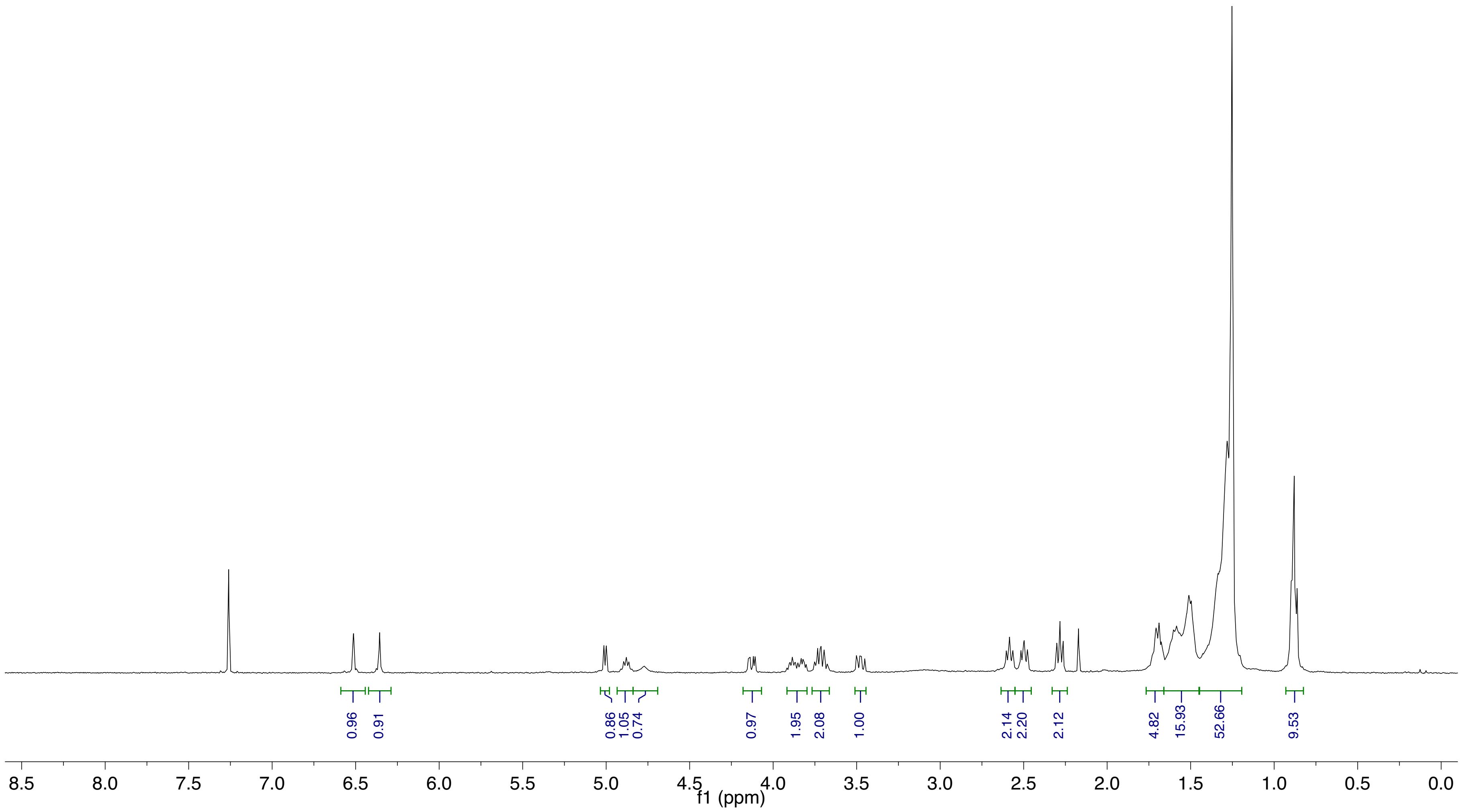

Supplementary Figure 32 – ^{1}H NMR ($CDCl_3$, 400 MHz) spectrum of compound **7a**.

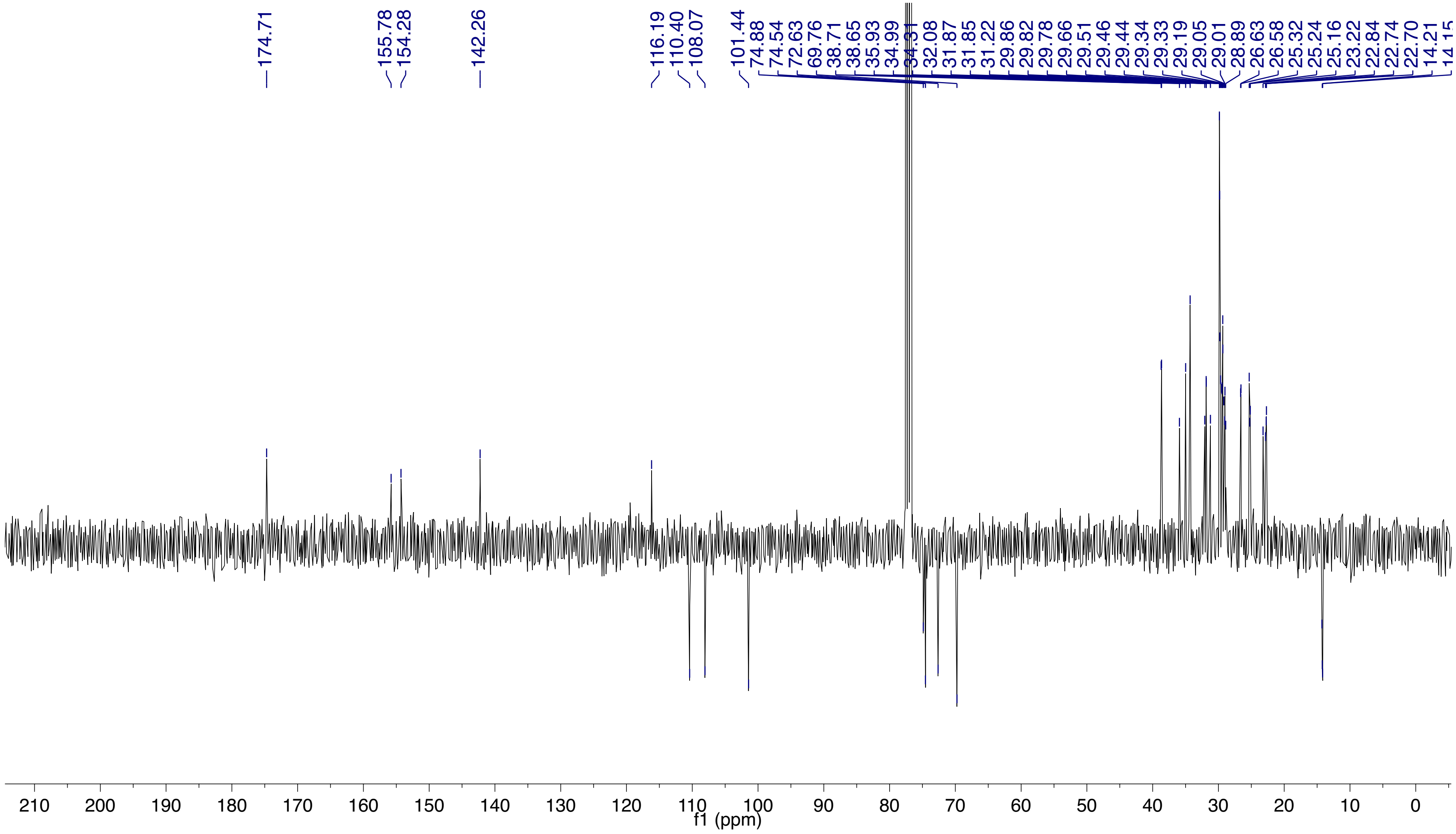

Supplementary Figure 33 – ^{13}C NMR (APT, $CDCl_3$, 100 MHz) spectrum of compound **7a**.

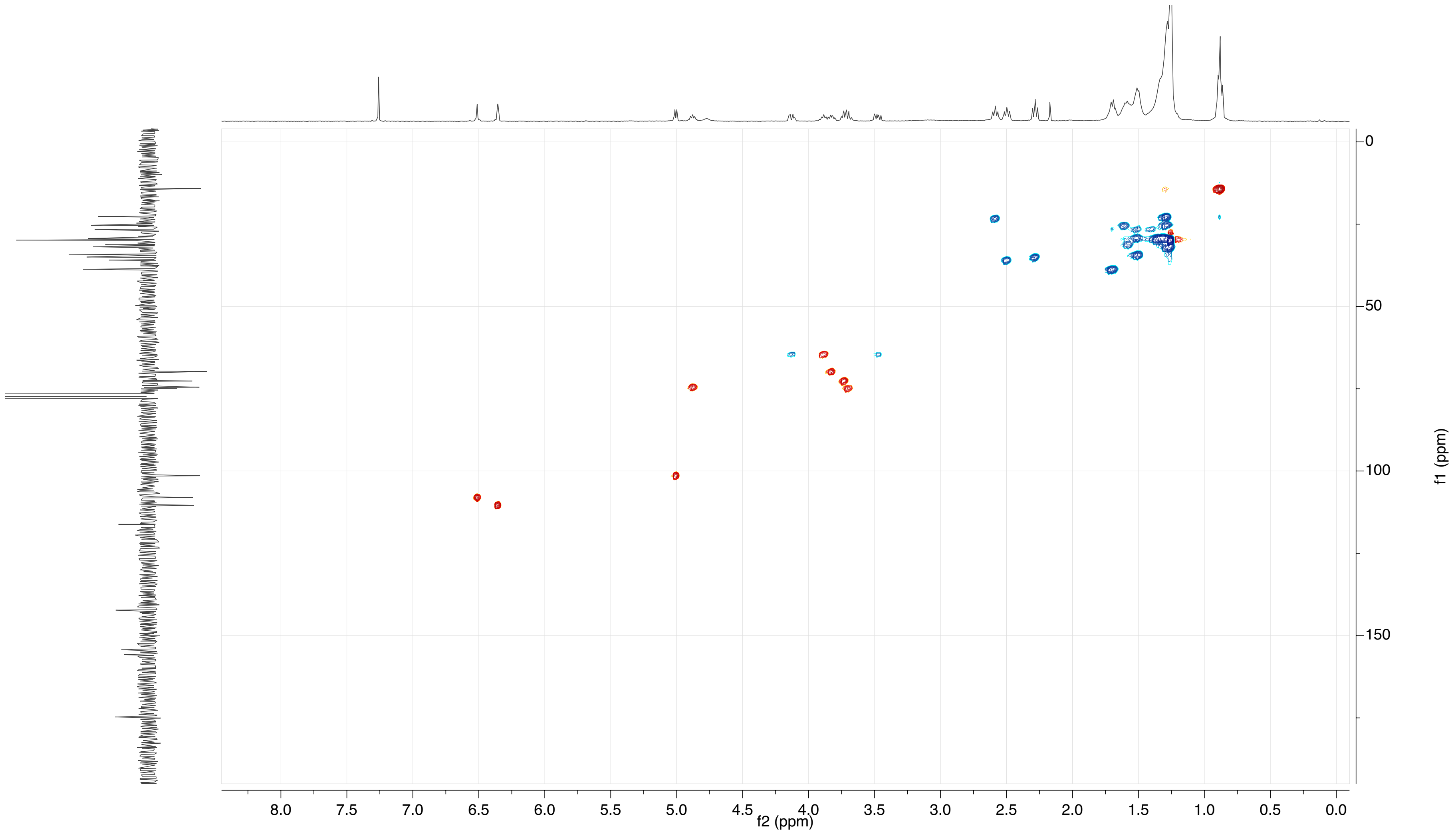

Supplementary Figure 34 – HSQC ($CDCl_3$, 400 MHz) spectrum of compound **7a**.

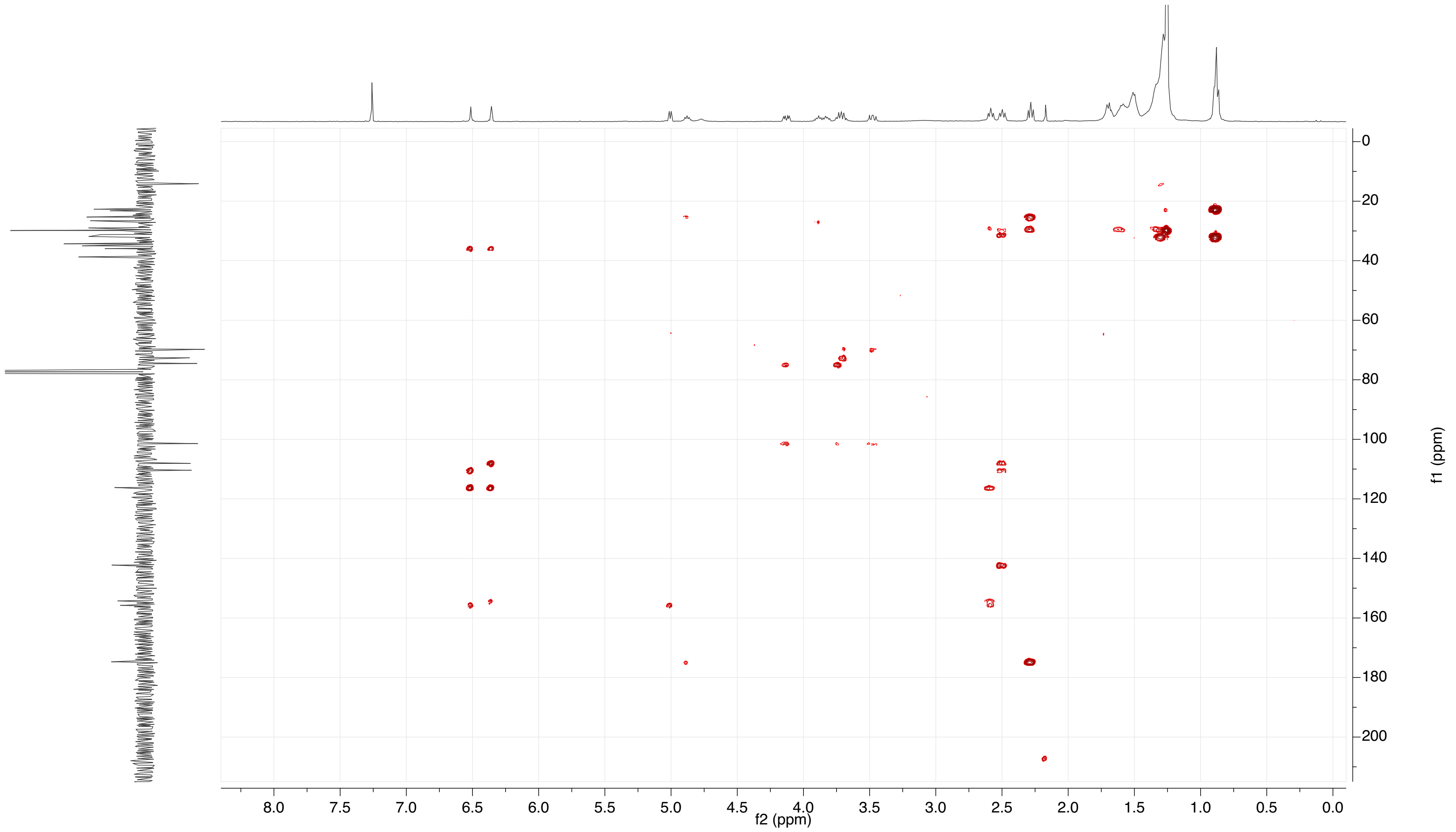

Supplementary Figure 35 – HMBC ($CDCl_3$, 400 MHz) spectrum of compound **7a**.

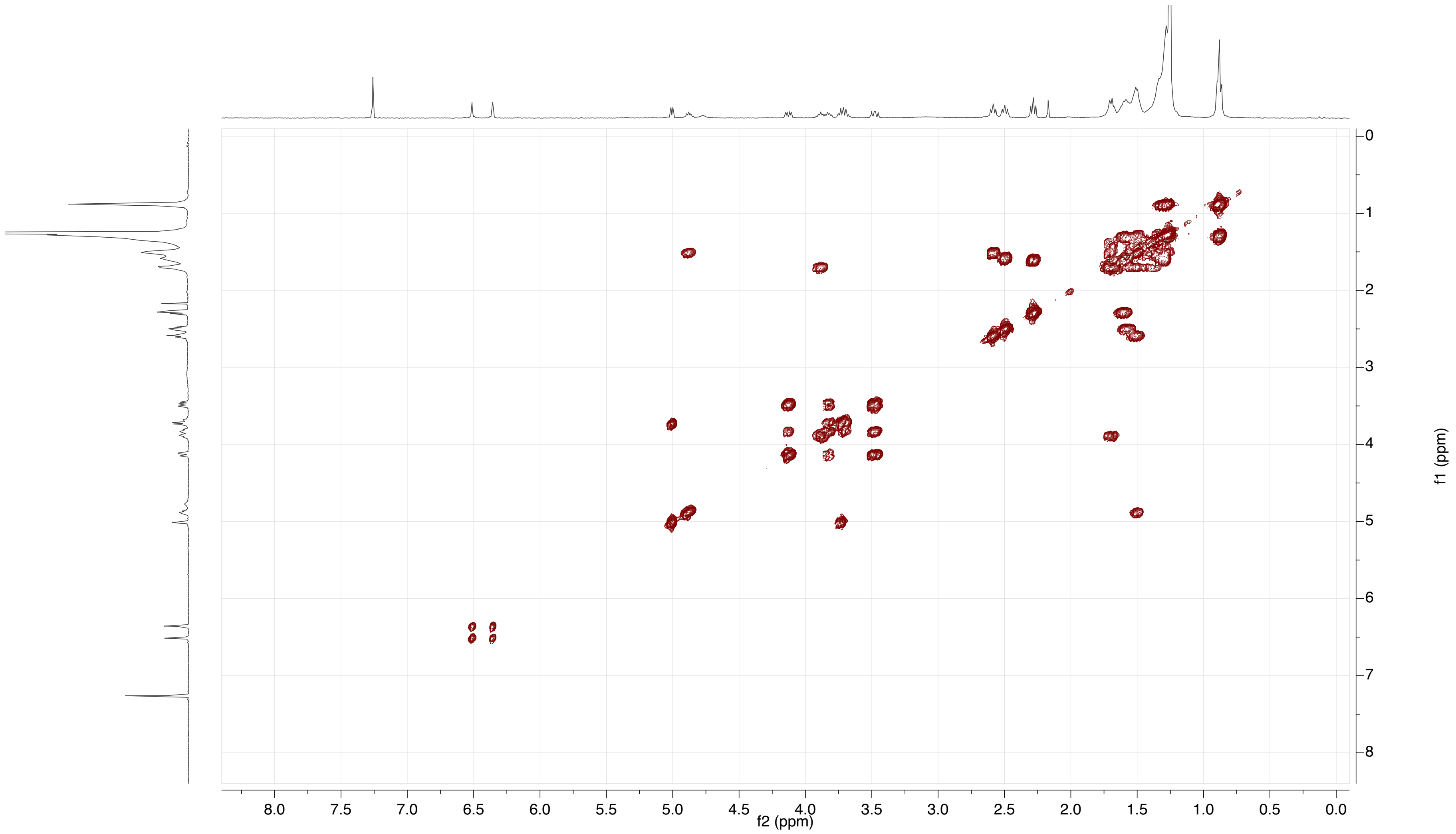

Supplementary Figure 36 – COSY ($CDCl_3$, 400 MHz) spectrum of compound **7a**.

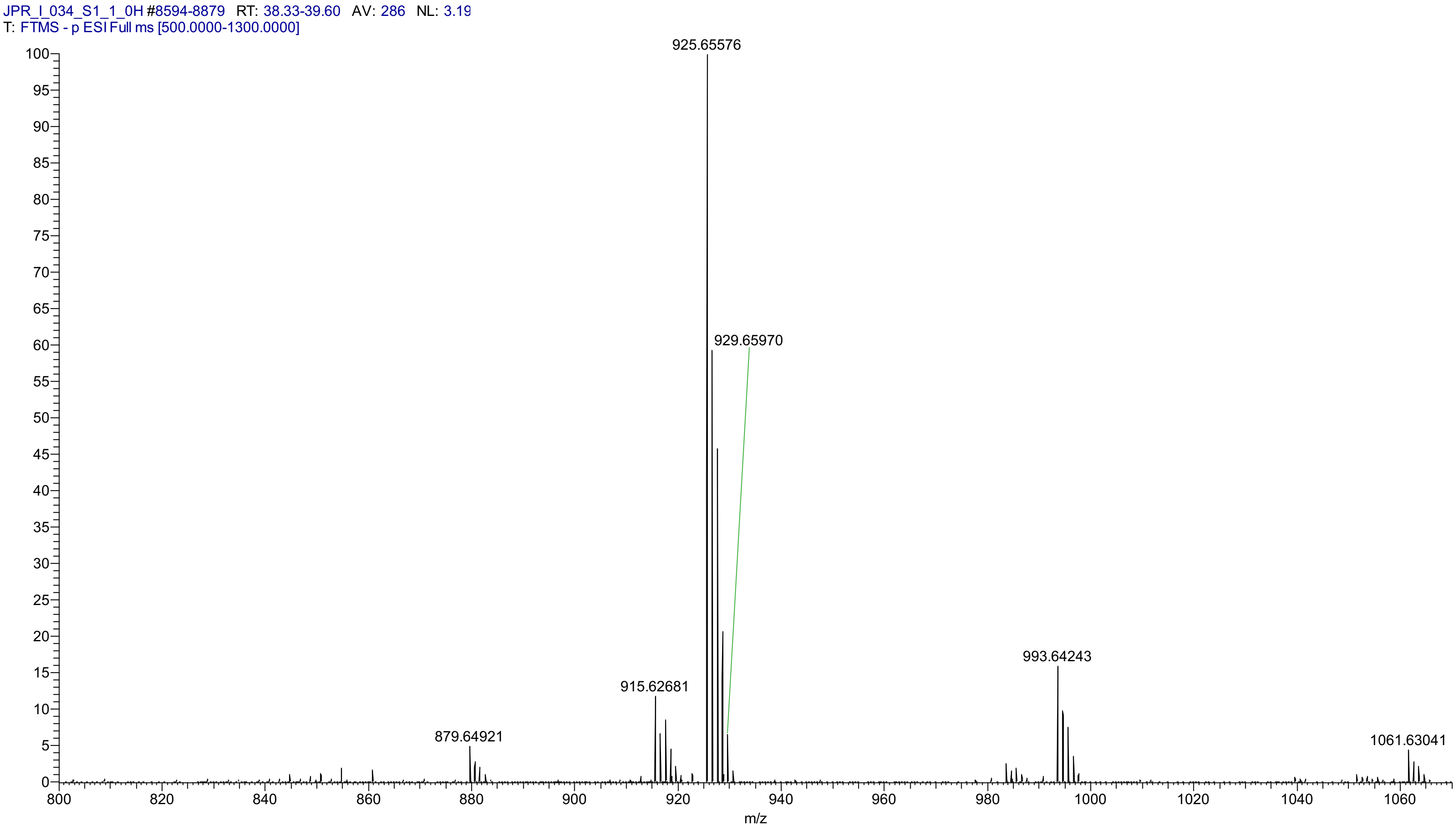

Supplementary Figure 37 – HRESIMS spectrum of compound **7a**.

Supplementary Table 4 – NMR Spectroscopic Data (^{1}H 600 MHz, ^{13}C 150 MHz, $CDCl_3$) for partially purified bartoloside A-29-yl palmitate (**7b**).

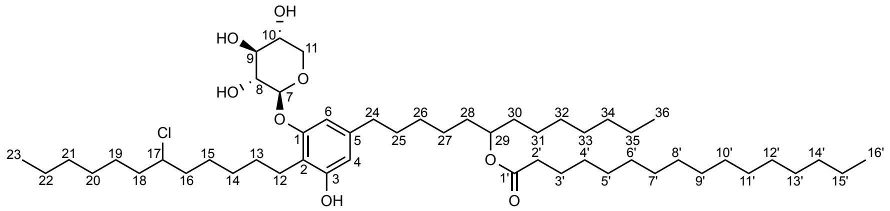

7b

position	δC	type	δH	mult, *J* (Hz)
1	155.6	C		
2	116.1	C		
3	155.5	C-OH		
4	110.5	CH	6.38	s
5	142.0	C		
6	107.8	CH	6.50	s
7	101.2	CH	5.04	d, 5.4
8	72.3	CH-OH	3.74	m
9	74.3	CH-OH	3.73	m
10	69.7	CH-OH	3.84	m
11	64.6	CH_2	4.13/3.49	dd, 12.0, 4.1/dd, 12.0, 7.6
12	23.5	CH_2	2.58	m
13	29.2	CH_2	1.51	m
14, 20, 26, 32, 33, 4'-13'	29.9-28.7	15 × CH_2	1.27-1.24	m
15a, 19a	25.4-25.3	2 × CH_2	1.61-1.60	m
15b, 19b			1.30-1.28	m
16a, 18a	34.4-34.1	2 × CH_2	1.51-1.50	m
16b, 18b			1.27-1.24	m
17	74.3	CH	4.89	m
21, 34, 14'	32.1-31.5	3 × CH_2	1.27-1.24	m
22, 35, 15'	22.9-22.7	3 × CH_2	1.30-1.28	m
23, 36, 16'	14.3-14.2	3 × CH_3	0.88	m
24	35.3	CH_2	2.51	t, 7.2
25	30.7	CH_2	1.58	m
27a, 31a	26.6-26.3	2 × CH_2	1.51-1.50	m
27b, 31b			1.41-1.40	m
28/30	38.7	2 × CH_2	1.69	m
29	64.5	CH-Cl	3.88	m
1'	174.5	C		
2'	35.0	CH_2	2.29	t, 7.5
3'	25.4	CH_2	1.61-1.60	m

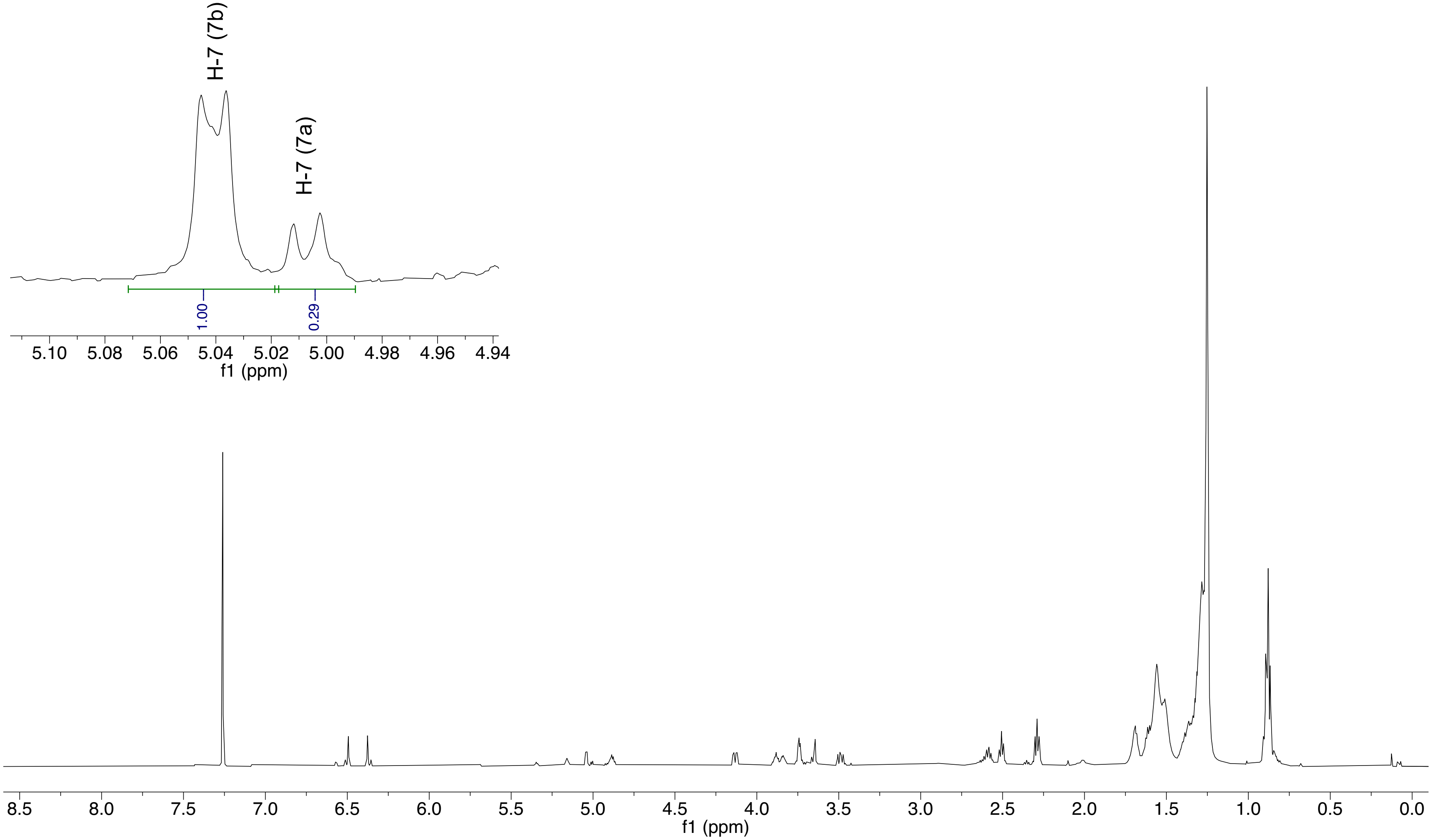

Supplementary Figure 38 – ^{1}H NMR ($CDCl_3$, 600 MHz) spectrum of partially purified compound **7b**. Insert shows anomeric proton (H-7) resonances for **7b** and **7a**, used to calculate relative abundance of the two compounds in the sample.

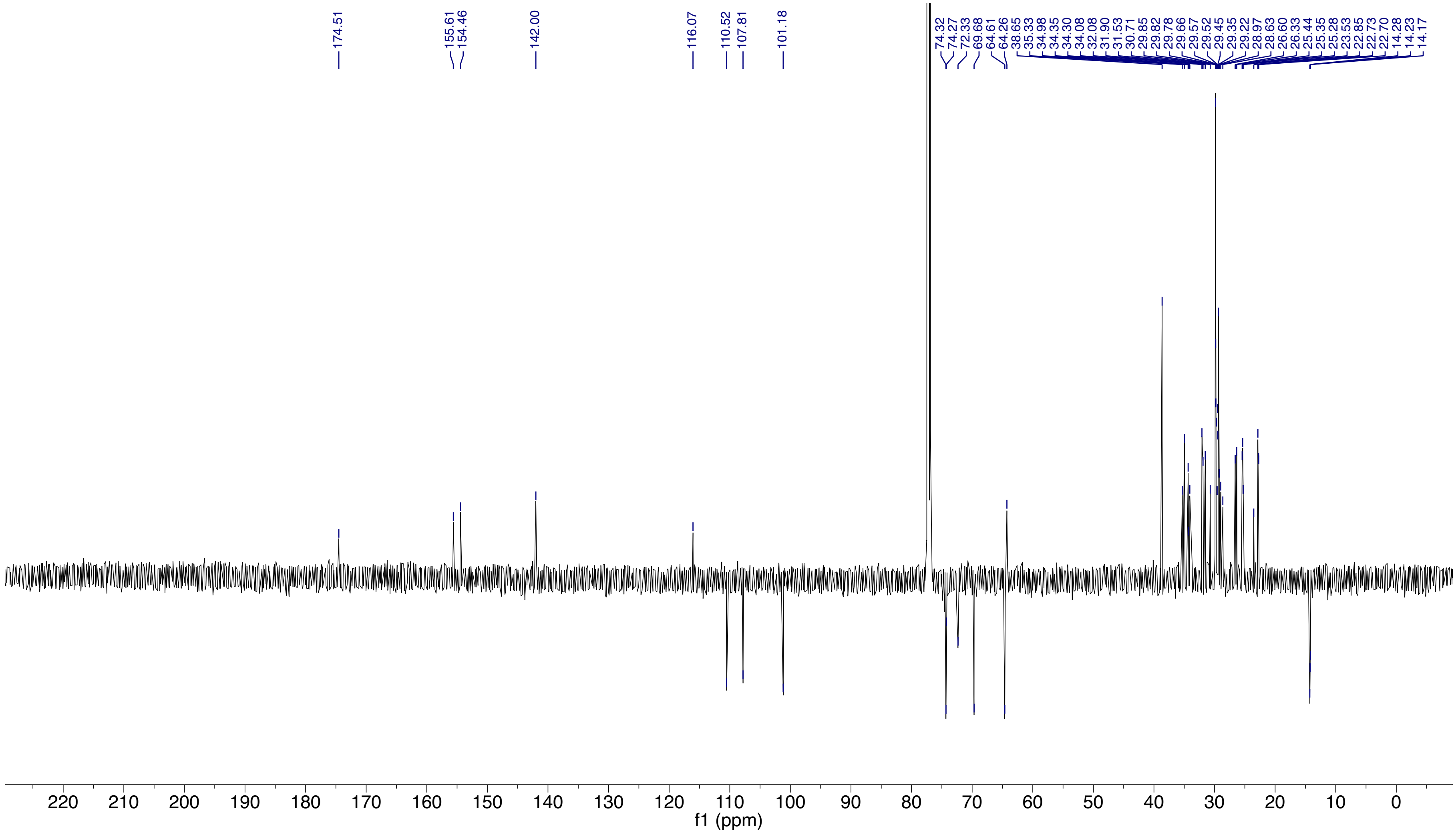

Supplementary Figure 39 – ^{13}C NMR (APT, $CDCl_3$, 150 MHz) spectrum of partially purified compound **7b**.

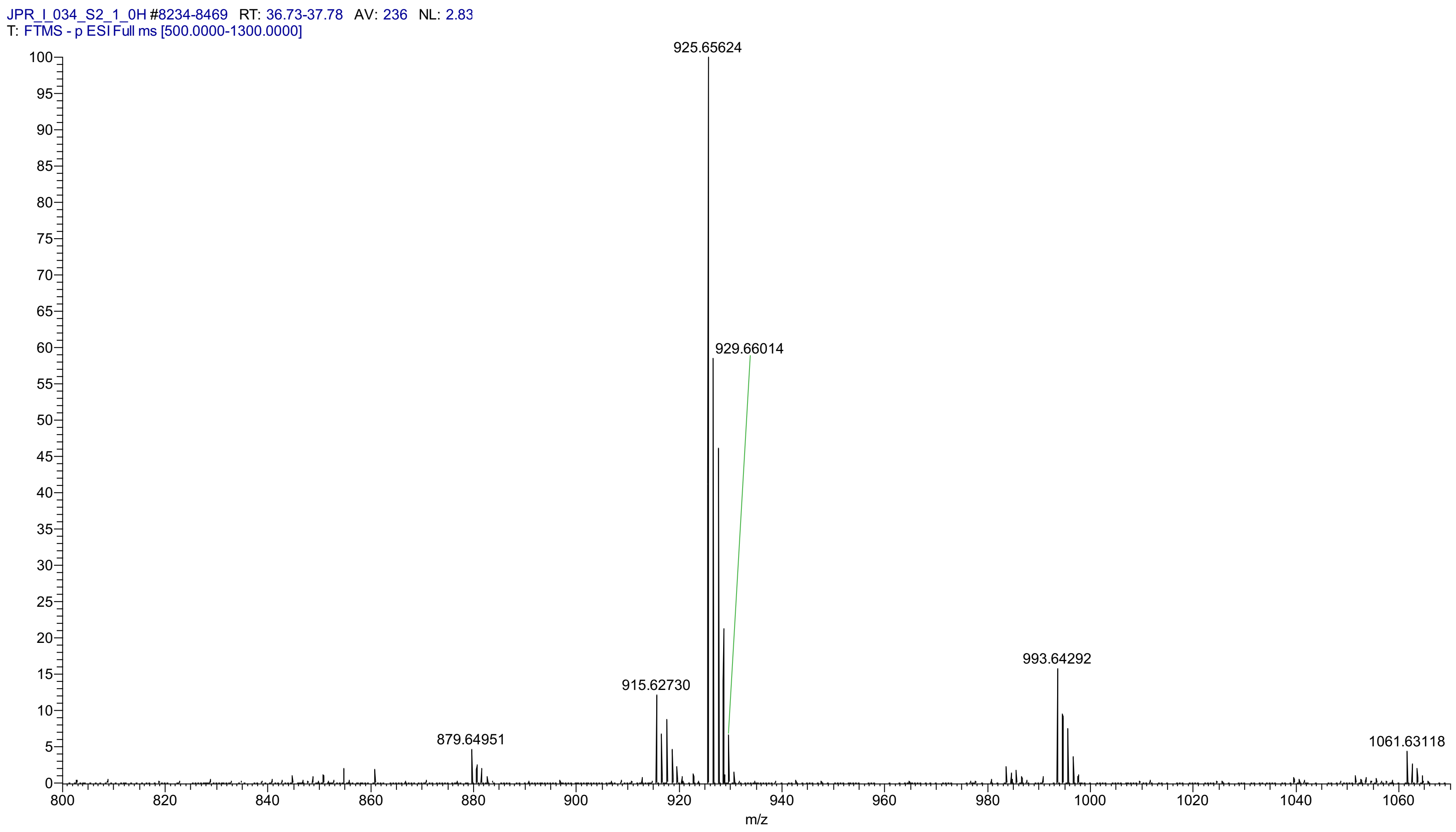

Supplementary Figure 40 – HRESIMS spectrum of compound **7b**.

Supplementary references

1. Leão, P. N. *et al.* Biosynthesis-Assisted Structural Elucidation of the Bartolosides, Chlorinated Aromatic Glycolipids from Cyanobacteria. *Angew. Chem. Int. Ed.* **54**, 11063–11067 (2015).
2. Afonso, T. B. *et al.* Bartolosides E–K from a Marine Coccoid Cyanobacterium. *J. Nat. Prod.* **79**, 2504–2513 (2016).
